# Integrated Multi-Omics Identifies Core Molecular Targets in Cerebral Venous Sinus Thrombosis-Induced Brain Injury

**DOI:** 10.3390/biomedicines14071594

**Published:** 2026-07-16

**Authors:** Xiaohong Qin, Haoran Lu, Zhibiao Chen, Yuxuan Wang, Jiang Chen, Xizhi Liu, Zilong Zhao, Jiaqi Zhou, Shuyue Tian, Rui Ding

**Affiliations:** 1 Department of Neurosurgery, Renmin Hospital of Wuhan University, Wuhan 430060, China; 2021283020213@whu.edu.cn (X.Q.); 2022283020096@whu.edu.cn (H.L.); chzbiao@126.com (Z.C.); yuxuan15@whu.edu.cn (Y.W.); 2023283020139@whu.edu.cn (X.L.); 2024283020125@whu.edu.cn (Z.Z.); 2025203020027@whu.edu.cn (J.Z.); 2024305231093@whu.edu.cn (S.T.); 2 Central Laboratory, Renmin Hospital of Wuhan University, Wuhan 430060, China; ddmm818521@163.com; 3 Department of Medical Imaging, Hanzhong Hospital of Traditional Chinese, Hanzhong 723000, China

**Keywords:** cerebral venous sinus thrombosis, multi-omics, single-cell sequencing, molecular docking, molecular dynamics, therapeutic targets, brain injury, rat model

## Abstract

**Background:** Cerebral venous sinus thrombosis (CVST) is a critical cause of brain injury and intracranial hypertension. However, its underlying molecular mechanisms remain poorly understood, limiting the development of targeted therapies. This study aims to systematically identify key molecular targets and signaling pathways involved in CVST-induced brain lesions using multi-omics approaches in a modified rat model of CVST. **Methods:** An optimized rat CVST model was established. Cortical tissues were collected from Sham-operated, 2-day post-CVST, and 7-day post-CVST groups for transcriptomic, proteomic, and single-cell transcriptomic sequencing. Bioinformatics analyses were performed to identify differentially expressed genes/proteins, followed by functional enrichment, protein–protein interaction network construction, and hub-gene screening. Further investigations included drug enrichment analysis, molecular docking, and molecular dynamics, as well as the prediction of competing endogenous RNA networks, transcription factor analysis, and expression profiling of potential edema-related therapeutic targets. **Results:** Multi-omics analyses revealed dynamic changes in gene and protein expression in the brain after CVST, along with associated pathways involved in immune inflammatory responses and tissue repair. Integrative analysis identified 12 core genes (*Cd44*, *Cd40*, *Sdc1*, *Myd88*, *Icam1*, *Stat3*, *Jak2*, *Ptgs2*, *Aldh1a1*, *Hspb1*, *Pxdn*, and *Casp3*). Single-cell RNA sequencing validated their expression and delineated cell-type specificity. Molecular docking hinted at the high binding potential of glucocorticoids such as dexamethasone and methylprednisolone to several core targets (*JAK2*, *PTGS2*, and *CD44*), with all docked complexes showing binding energies below −8.2 kcal/mol. Further molecular dynamics simulations indicated that methylprednisolone forms a stable complex with CD44, driven primarily by van der Waals and electrostatic interactions. Additionally, dynamic levels of several potential edema-related targets (*Kcnn4*, *Piezo1*, *Trpv4*, and *Atp1a2*) were observed. **Conclusions:** In summary, by applying integrated multi-omics profiling to a modified rat model, this study systematically mapped the molecular landscape of CVST-induced brain injury. A number of candidate targets and signaling pathways emerged from our analysis, along with several compounds of potential therapeutic interest. Collectively, these results provide a basis for further investigation into the mechanisms underlying CVST and for the design of novel treatment approaches.

## 1. Introduction

Ischemic stroke is a leading cause of death and disability worldwide [1]. Based on the affected vessel, it includes arterial ischemic stroke and venous stroke. In the latter—also termed cerebral venous thrombosis—the process begins with blood clots inside the cerebral veins, which then block both venous drainage and the circulation of cerebrospinal fluid [2,3]. This form of stroke, known as cerebral venous sinus thrombosis (CVST), is most commonly seen in young adults, particularly in postpartum women [4,5]. Although headache is the most frequent presenting symptom, the condition’s variable presentation often results in diagnostic delays and high misdiagnosis rates (up to 73%) [6]. In severe cases, mortality can reach 34.2%, and those who survive often face lasting neurological deficits that affect their ability to work [7,8].

Research into arterial stroke has been extensive [1,9]. The same cannot be said for venous stroke, where progress has lagged, and with it, efforts to catch the disease early or develop targeted therapies. Multi-omics tools (transcriptomics, proteomics, single-cell sequencing) now give us a sharper way to dissect disease biology. Already, these approaches have turned up critical pathways in several conditions, including migraine [10], psoriasis [11], and ischemic stroke [12]. Single-cell transcriptomics adds another dimension by revealing cellular heterogeneity beyond bulk sequencing averages [13,14]. Garcia-Bonilla et al. demonstrated dynamic immune cell changes after stroke using this approach [15]. However, existing CVST animal models are often time-consuming, poorly reproducible, and fail to mimic clinical recanalization [16].

Despite the clinical significance of CVST and the severe neurological consequences it entails, the molecular mechanisms driving post-CVST brain injury remain largely undefined. In particular, the molecular targets that mediate post-CVST neuroinflammation, cerebral edema, and tissue repair have not been systematically characterized, and the potential for repurposing existing anti-inflammatory drugs in this context remains unexplored. We, therefore, hypothesized that integrating multi-omics profiling (transcriptomics, proteomics, and single-cell transcriptomics) with computational pharmacology (molecular docking and molecular dynamics) would identify a tractable panel of hub genes and pathways that could serve as therapeutic targets and would reveal candidate repurposable drugs for CVST.

To test this hypothesis, we employed an optimized rat CVST model with integrated transcriptomic, proteomic, and single-cell analyses to identify molecular targets and pathways linked to post-CVST brain injury. We also performed drug enrichment analysis, molecular docking, and molecular dynamics, as well as predicting competing endogenous RNA (ceRNA) networks and transcription factors. Additionally, we identified potential targets involved in cerebral edema. These findings lay a foundation for developing targeted therapies for CVST-induced brain injury.

## 2. Materials and Methods

### 2.1. Experimental Animals

Adult male Sprague-Dawley rats (8–10 weeks, 250–300 g) were purchased from Hunan Slike Jingda (Changsha, China). Animals were housed under SPF conditions (22 ± 2 °C, 50–60% humidity, 12 h light/dark) with free access to food/water. All procedures were approved by the Animal Welfare Ethics Committee of Wuhan University Renmin Hospital (Approval No. WDRM-DW (Fu) 20250303B, 7 March 2025) and followed the Animal Research: Reporting of In Vivo Experiments (ARRIVE) guidelines.

### 2.2. Establishment of a Modified Rat CVST Model

Based on the method reported by Xiao et al. [17] with modifications, we developed an improved model of superior sagittal sinus (SSS) thrombosis in rats, i.e., a CVST model, by combining vascular clipping with ferric chloride (FeCl_3_) and thrombin induction.

Anesthesia was induced in male SD rats via intraperitoneal injection of 1% sodium pentobarbital (40 mg/kg). Once fully anesthetized, each animal was placed in a stereotaxic frame (RWD Life Science, Shenzhen, China). The scalp was shaved, disinfected, and then opened with a midline incision. After blunt dissection of the periosteum, the bregma and lambda were clearly exposed. These landmarks guided identification of the underlying SSS. Using a high-speed cranial drill (Shanghai Yuyan Scientific Instruments, Co., Ltd., Shanghai, China) with continuous saline irrigation for cooling, the bone overlying the SSS between the bregma and lambda was carefully removed to create a window roughly 10 mm × 4 mm. Drilling speed and pressure were carefully controlled to avoid thermal injury and dura penetration.

After full exposure of the SSS, miniature aneurysm clips (Meike Innovation Technology Co., Ltd., Beijing, China; 5 mm length) were temporarily applied at the levels of the bregma and lambda to block SSS blood flow. A segment of 3-0 surgical suture (about 5.5 mm long) soaked in 40% FeCl_3_ solution (Sigma-Aldrich, Shanghai, China) was applied in darkness onto the exposed SSS surface for 5 min. The surgical area was then gently rinsed at least five times with 37 °C saline to remove residual FeCl_3_. Thrombin (Solarbio, Beijing, China, T8021; 100 U/100 µL) was then delivered slowly into the clipped segment of the SSS using a microsyringe (Hamilton, Shanghai, China), and we waited until a visible clot had formed.

Once thrombosis was established, the aneurysm clips were taken off, and the surgical field was flushed three times with warm saline. The scalp incision was closed in layers after local disinfection. Right after surgery, each rat received 2 mL of warm 5% glucose via intraperitoneal injection to prevent dehydration and was placed on a heating pad at 37 °C until fully awake. Once conscious, the animals went back to their home cages, where they had free access to food and water. Neurological status and wound healing were checked every day. Sham-operated rats underwent craniotomy with bone removal and temporary application of aneurysm clips without FeCl_3_ or thrombin induction.

### 2.3. Laser Speckle Blood Flow Imaging

Blood flow in the SSS and cortex was monitored using a laser speckle blood flow imaging system (SIM BFI ZOOM, Wuhan, China). Baseline imaging was performed on Sham rats, then repeated at 48 h post-surgery in the same region of interest (ROI) after CVST induction. All data were analyzed using the system’s software.

### 2.4. 2,3,5-Triphenyltetrazolium Chloride (TTC) Staining

To assess ischemic injury, rats were perfused with ice-cold saline; brains were removed, rinsed, and hardened at −20 °C for 15 min. Coronal sections (2 mm) were stained with 2% TTC (37 °C, 20 min, in the dark), fixed in 4% paraformaldehyde for 6 h, and then imaged.

### 2.5. Experimental Groups and Sample Preparation

Twenty-four rats were randomly assigned to Sham (n = 6), CVST 2d (n = 6), and CVST 7d (n = 6) groups. From each, three samples were used for transcriptomic and proteomic analyses; three Sham and three CVST 2d samples underwent single-cell sequencing.

At 48 h or 7 days, rats were anesthetized (1% pentobarbital, 40 mg/kg, i.p.), perfused with saline, and cortices dissected on ice. Based on the literature [18,19], the selected brain region was primarily the injured cerebral cortex in the parietal lobe. Tissue (~50 mg) was snap-frozen (−80 °C) for omics or placed in preservation solution for single-cell RNA-seq.

### 2.6. Transcriptomic Analysis

Cortical samples (n = 3/group) underwent RNA extraction and library construction (RIN > 7.0) using an Illumina-compatible kit (Supplementary Materials and Methods 1). Sequencing was performed on a NovaSeq 6000 (150 bp paired-end). Raw data were processed on the Majorbio Cloud platform [20] with fastp [21] for trimming and filtering using the following parameters: a sliding window of 4 bp with a minimum mean quality score of 20, removal of reads shorter than 50 bp, and adapter trimming. Reads containing more than 5% ambiguous bases (N) were discarded. Clean reads were aligned to Rnor_6.0 reference genome using HISAT2 (v2.2.1) [22] with default settings; uniquely mapped reads were defined as those mapping to a single genomic location with ≤2 mismatches. Transcript assembly and quantification were performed with StringTie (v3.0.3) [23]. Expression levels were normalized to transcripts per million (TPM) using RSEM (v1.3.3) [24]. Differentially expressed genes (DEGs) (|log_2_FC| ≥ 1, FDR < 0.05) were identified using DESeq2 (v1.48.1) [25]. Gene Ontology (GO), Kyoto Encyclopedia of Genes and Genomes (KEGG), and Reactome pathway enrichment employed the hypergeometric test with Benjamini–Hochberg (BH) correction (Padj < 0.05), using Goatools (v1.5.1), SciPy (https://scipy.org/, accessed on 1 July 2025), and ReactomePA (v1.54.0).

### 2.7. Proteomic Analysis

Cortical samples (n = 3/group) underwent protein extraction, digestion, and liquid chromatography–mass spectrometry in data-independent acquisition (DIA) [26] mode on a TimTOF Pro 2 system (Supplementary Materials and Methods 2, Majorbio, Shanghai, China). Raw data were processed on Majorbio Cloud platform. Proteins were identified and quantified using DIA-NN (v1.8.1) against Rnor_6.0 protein database, with a false discovery rate <1% at both peptide and protein levels. Only proteins identified by at least one unique peptide were retained. Missing values were imputed using the k-nearest neighbor (k-NN) method with k = 10. Differentially expressed proteins (DEPs) were tested using Student’s *t*-test (*p* < 0.05, fold change >1.2 or <0.83). For proteomic data, we applied a nominal *p* < 0.05 threshold combined with a stringent fold-change cutoff, rather than FDR correction. This approach was chosen because: (i) proteomic datasets typically have lower dynamic range and signal-to-noise ratio compared to transcriptomic data, and FDR correction can be overly conservative; (ii) the fold-change cutoff of 1.2 provides an additional filter to prioritize biologically meaningful differences while reducing false positives; and (iii) this is an exploratory study aimed at hypothesis generation, and the identified DEPs were further validated through orthogonal approaches including single-cell transcriptomics. Nevertheless, we acknowledge that this approach does not control for multiple comparisons, which is noted as a limitation (see Section 4). The DEPs were then subjected to GO/KEGG/Reactome enrichment using the hypergeometric test with BH correction (Padj < 0.05).

### 2.8. Clustering Analysis

Hierarchical clustering was performed on selected genes/proteins using Euclidean distance and the average linkage method as implemented in the hclust() function in R (version 4.1.0). Based on TPM expression matrices, the genes and proteins were grouped into ten sub-clusters for functional annotation.

### 2.9. Sample Correlation, Principal Component Analysis, and Co-Expression Analysis

To evaluate reproducibility and sample relationships, Pearson correlation was computed for all sample pairs; outliers were excluded. Principal component analysis (PCA) was used to visualize group separation. For gene co-expression, Spearman correlation (≥0.8, BH-adjusted *p* ≤ 0.05) was calculated for all gene pairs.

### 2.10. Integrated Transcriptomic and Proteomic Analysis

To identify core regulatory molecules, DEGs and DEPs were intersected to obtain candidate genes. A protein–protein interaction (PPI) network was constructed using STRING (confidence ≥ 0.400) and visualized in Cytoscape (v3.8.0). The MCODE plugin was used to identify densely connected sub-modules (parameters: Degree Cutoff = 2, Node Score Cutoff = 0.2, K-Core = 2, Max. Depth = 100). CytoHubba ranked genes using 11 algorithms; the top 100 from each were intersected to yield core genes as candidate targets.

### 2.11. Single-Cell Transcriptomic Sequencing Analysis

Cortical samples from Sham and CVST 2d groups (n = 3 each) were dissociated into single-cell suspensions (viability > 85%) using Singleron protocols. Libraries were prepared with the GEXSCOPE^®^ kit (Supplementary File) and sequenced on an Illumina NovaSeq 6000 (Supplementary Materials and Methods 3). Raw data were processed with CeleScope (Singleron, Nanjing, China). Barcodes/UMIs were extracted from Read 1; adapters/poly-A tails trimmed from Read 2. Reads were aligned to mRatBN7.2 using STARsolo, and uniquely mapped reads were merged to generate an expression matrix. Data were analyzed on CeleLens Cloud with default parameters (quality filtering, normalization, clustering, etc.). Automatic cell annotations were manually verified using marker genes. Downstream analyses were performed on the platform.

### 2.12. Drug Enrichment Analysis, Molecular Docking, and Molecular Dynamics

Compounds targeting the feature genes were screened using the Drug–Gene Interaction Database (DGIdb) and the Drug Signatures Database (DSigDB). Drug enrichment analysis (hypergeometric test, adjusted *p* ≤ 0.05) identified candidates for molecular docking. The eight compounds selected for docking (dexamethasone, methylprednisolone, hydrocortisone, mometasone furoate, simvastatin, thalidomide, ibrutinib, and WP1066) were chosen based on the following criteria: (i) they are either FDA-approved or have well-documented safety profiles in clinical use; (ii) they were predicted to interact with one or more of the five core proteins (CD44, ICAM1, JAK2, MYD88, PTGS2) based on database queries; and (iii) they showed initial binding energies below −8.0 kcal/mol in preliminary docking screens. Among these, the glucocorticoids (dexamethasone, methylprednisolone, hydrocortisone, mometasone furoate) were prioritized for deeper molecular dynamics analysis because of their known anti-inflammatory properties and clinical relevance to neuroinflammation and thrombosis.

Three-dimensional (3D) structures of drugs (PubChem) and proteins (RCSB PDB) were used. Blind docking was performed with CB-Dock2, refined with AutoDock Vina (v1.2.5). Complexes with binding energy <−8.0 kcal/mol and ≥1 hydrogen bond were considered stable. Interactions were visualized using Discovery Studio 2019.

We started with the methylprednisolone–CD44 complex obtained from molecular docking and ran all-atom molecular dynamics simulations using AMBER 24. We assigned charges to the small molecule using the AM1-BCC method and described it with the GAFF2 force field; for the protein, we used ff14SB. We put the system inside a truncated octahedral box with TIP3P water, making sure to leave at least 10 Å of solvent around the protein. To balance the net charge, we added Na^+^ and Cl^−^ ions.

Before the simulation could start, we ran energy minimization—2500 steps of steepest descent followed by another 2500 of conjugate gradient. Next came a gradual warm-up from 0 K to 298.15 K, which took 200 ps. After that, we performed 500 ps of NVT equilibration, then another 500 ps under NPT conditions. The production run lasted 50 ns under NPT conditions with periodic boundaries. We set a 10 Å cutoff for non-bonded interactions, used Particle Mesh Ewald to handle long-range electrostatics and applied SHAKE to keep bonds involving hydrogen in check. Temperature was regulated with Langevin dynamics (collision frequency γ = 2 ps^−1^), pressure was held at 1 atm, and we used a 2 fs time step, saving coordinates every 10 ps. Binding free energies were calculated with the MM/GBSA method, using snapshots from the final 10 ns of the trajectory (40–50 ns). The binding energy was estimated as follows:

ΔG_bind = ΔG_complex − (ΔG_receptor + ΔG_ligand) = ΔE_VDW + ΔE_elec + ΔG_GB + ΔG_SA

In this equation, ΔE_VDW and ΔE_elec stand for van der Waals and electrostatic contributions, while ΔG_GB and ΔG_SA capture the polar and non-polar parts of solvation. From the simulation trajectories, we also calculated several key metrics: root-mean-square deviation (RMSD, a measure of structural drift over time), root-mean-square fluctuation (RMSF, indicating residue-wise flexibility), radius of gyration (Rg, reflecting compactness), hydrogen bond counts, and solvent-accessible surface area (SASA, a measure of exposure). The free energy landscape was constructed based on the joint distribution of RMSD and Rg.

### 2.13. ceRNA Regulatory Network and TF Prediction for Feature Genes

miRNA binding sites on feature genes were predicted using miRanda, miRDB, miRTarBase, and TargetScan; only miRNAs common to all four were retained. SpongeScan predicted lncRNAs targeting these miRNAs to construct a ceRNA network, visualized in Cytoscape [27]. TRRUST predicted upstream TFs regulating the feature genes.

### 2.14. Statistical Analysis

Statistical methods for each omics dataset are detailed in corresponding sections. For multi-group comparisons, one-way ANOVA was used (*p* < 0.05). All analyses were performed using R or the respective platforms.

## 3. Results

### 3.1. Establishment and Validation of an Improved Rat CVST Model

**Figure 1:**
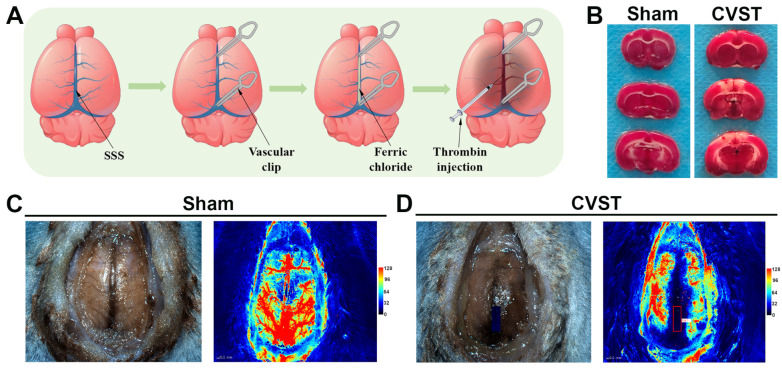
Improved rat CVST model. (**A**) Schematic of the CVST model construction. (**B**) Representative TTC-stained brain sections from Sham and CVST 2d groups. (**C**,**D**) Laser speckle blood flow images of the cerebral cortex in Sham (**C**) and CVST 2d (**D**) rats. These data confirm that our modified model successfully induces SSS thrombosis, cerebral infarction, and significant cortical hypoperfusion.

We successfully established an improved model of rat CVST. A schematic of the model construction procedure is presented in Figure 1A. To evaluate model efficacy, we first performed TTC staining on brain tissue from Sham and CVST 2d rats. The results revealed prominent infarct areas in the cerebral cortex of CVST 2d rats compared with Sham controls (Figure 1B). To quantitatively monitor local cerebral blood flow, we next employed laser speckle contrast imaging. Relative to Sham animals, cortical blood flow was markedly reduced in CVST 2d rats (Figure 1C,D). Together, these findings demonstrate that our modeling approach reliably induces CVST and leads to well-defined ischemic brain injury, indicating good reproducibility and robustness of the model.

### 3.2. Quality Control of Transcriptomic Sequencing Data

Transcriptomic sequencing of nine samples (n = 3/group) generated 58.5 Gb of clean data (≥5.88 Gb/sample). The sequencing error rate was <0.02%, Q20 > 99.0%, Q30 > 95.4%, and GC content ~50% (Supplementary Table S1). The average alignment rate averaged was 97.5%, with uniquely mapped reads >95.0% and multiple mapped reads <2.6% (Supplementary Table S2). Coverage distribution showed no 5′/3′ bias (Supplementary Figure S1A), and violin plots confirmed high intra-group expression consistency (Supplementary Figure S1B). These metrics confirmed that the data were suitable for downstream analysis.

### 3.3. Transcriptome-Wide Gene Expression Differences and Inter-Group Relationships

**Figure 2:**
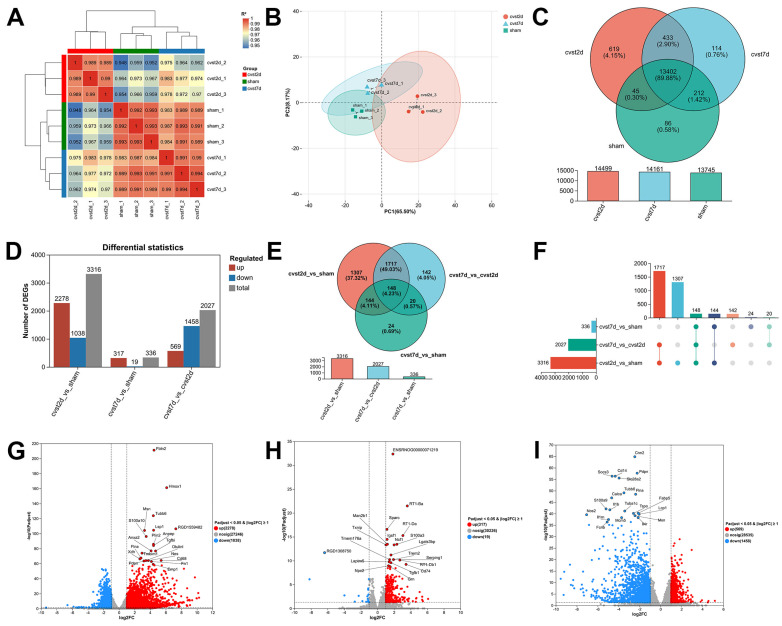
Differential transcriptomic analysis. (**A**) Inter-sample correlation heatmap. (**B**) Principal component analysis plot. (**C**) Venn diagram of expressed genes across the three groups. (**D**) Bar plot showing the number of differentially expressed genes. (**E**,**F**) Venn diagram (**E**) and UpSet plot (**F**) of DEGs. (**G**–**I**) Volcano plots of differentially expressed genes (corresponding to CVST 2d vs. Sham (**G**), CVST 7d vs. Sham (**H**), and CVST 7d vs. CVST 2d (**I**) comparisons). The acute phase (CVST 2d) exhibits far more transcriptional changes than the subacute phase, with thousands of genes altered, indicating a robust and dynamic cerebral transcriptomic response to CVST.

Inter-sample correlation (Pearson > 0.989) and PCA confirmed high intra-group reproducibility and clear separation of Sham, CVST 2d, and CVST 7d groups (Figure 2A,B). Expressed genes numbered 13,745 (Sham), 14,499 (CVST 2d), and 14,161 (CVST 7d), with 13,402 shared (Figure 2C; Supplementary Table S3). DEG analysis identified 3316 genes (2278 up, 1038 down) in CVST 2d vs. Sham; 336 genes (317 up, 19 down) in CVST 7d vs. Sham; and 2027 genes (569 up, 1458 down) in CVST 7d vs. CVST 2d (Figure 2D; Supplementary Tables S4–S6). Collectively, 3502 non-redundant DEGs were identified across comparisons, with distributions shown in Venn and UpSet plots (Figure 2E,F). Volcano plots display the top 20 most significantly altered genes for each comparison (Figure 2G–I).

### 3.4. Functional Annotation and Enrichment Analysis of DEGs

To systematically elucidate the molecular mechanisms underlying CVST progression, we performed GO, KEGG, and Reactome functional annotation and enrichment analyses on the DEGs from the three comparison sets: CVST 2d vs. Sham, CVST 7d vs. Sham, and CVST 7d vs. CVST 2d.

GO annotation results revealed that DEGs in all three comparisons were predominantly enriched in the biological process category, particularly in cellular processes, biological regulation, response to stress, and metabolic processes (Supplementary Figure S2A–C). GO enrichment analysis showed stage-specific patterns. Acute-phase (CVST 2d vs. Sham) DEGs were enriched in stress response, signal transduction, and immune regulation (Supplementary Figure S2D). Subacute-phase (CVST 7d vs. Sham) DEGs clustered in immune processes and response regulation (Supplementary Figure S2E). Recovery-phase (CVST 7d vs. CVST 2d) DEGs were enriched in development and multicellular organismal regulation (Supplementary Figure S2F).

**Figure 3:**
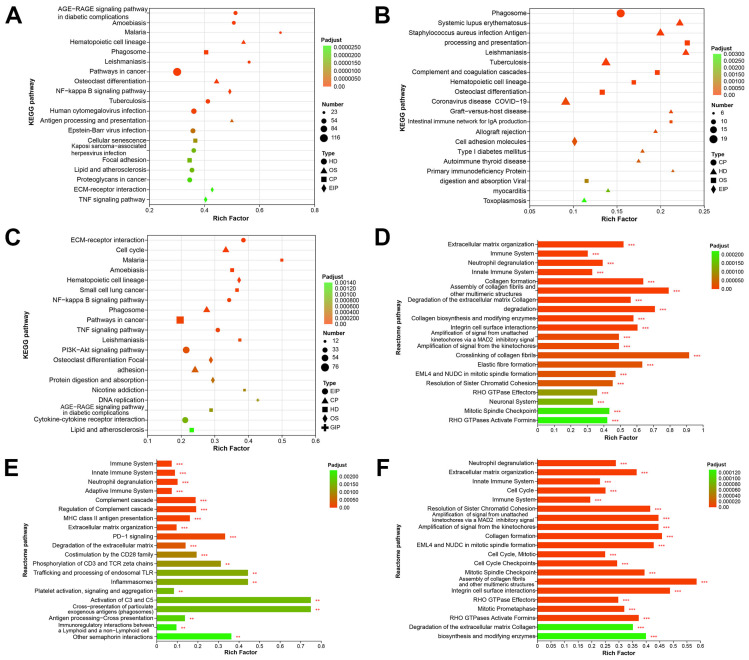
Functional enrichment analysis of DEGs. (**A**–**C**) Bubble plots of KEGG pathway enrichment. (**D**–**F**) Bar plots of Reactome pathway enrichment (adjusted *p* < 0.05). Significance levels are indicated as follows: *** for Padj < 0.001 and ** for Padj < 0.01. These data reveal a temporal progression from acute inflammation (CVST 2d) to adaptive immunity (CVST 7d) and then to tissue remodeling (recovery phase), with PI3K-Akt, ECM, and immune pathways consistently involved.

KEGG analysis revealed dynamic pathway alterations. Acute-phase pathways included focal adhesion, ECM-receptor interaction, TNF, NF-κB, and phagosome (Figure 3A). The subacute phase converged on phagosome, antigen presentation, cell adhesion molecules, and complement/coagulation cascades, plus autoimmune-related pathways (Figure 3B). Recovery phase featured ECM-receptor interaction, cell cycle, DNA replication, and PI3K-Akt signaling (Figure 3C).

Reactome analysis refined these findings. Acute phase: ECM organization (degradation), neutrophil degranulation, innate immunity (Figure 3D). Subacute phase: complement, adaptive immunity, MHC II presentation, inflammasome (Figure 3E). Recovery phase: cell cycle (mitosis), collagen formation, ECM synthesis (Figure 3F).

Integrating results from the three enrichment analyses revealed a temporal progression: acute inflammation → subacute refined immunity → recovery-phase tissue remodeling and proliferation. PI3K-Akt, ECM organization, and immune pathways were consistently implicated throughout CVST pathogenesis and repair.

### 3.5. Temporal Clustering and Functional Module Analysis of DEGs

**Figure 4:**
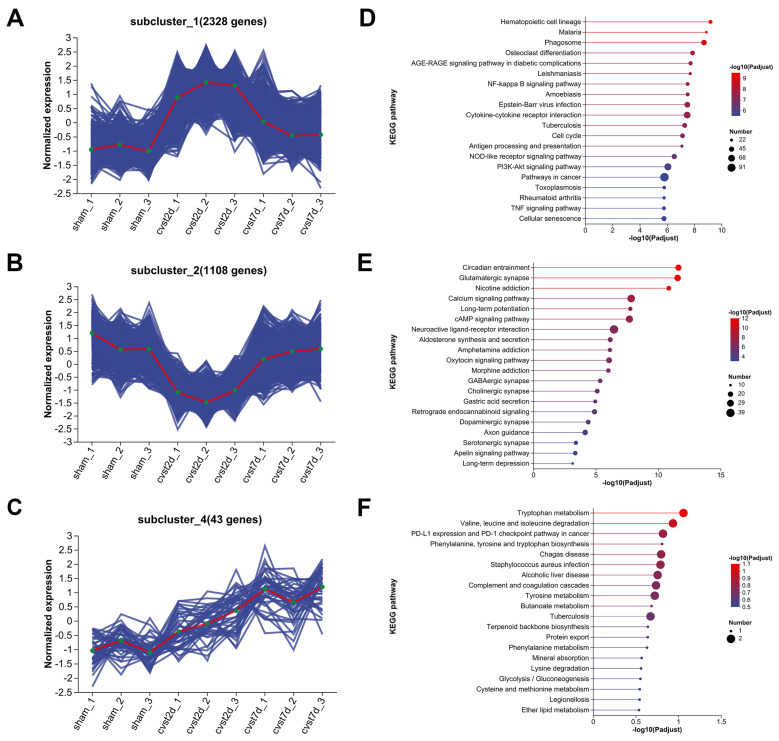
Clustering and functional enrichment analysis of DEGs. (**A**–**C**) Temporal expression trend plots illustrating the mean expression trajectories of sub-cluster 1 (2328 genes), sub-cluster 2 (1108 genes), and sub-cluster 4 (43 genes) across Sham (**A**), CVST 2d (**B**), and CVST 7d (**C**) groups. Lines represent individual gene expression trends; green dots show the mean expression of all genes per sample; the red line indicates the overall mean trend for each sub-cluster. (**D**–**F**) Bar plots of KEGG pathway enrichment for the three sub-clusters (adjusted *p* < 0.05). Bar length represents the enrichment factor; color denotes significance level. These data identify three distinct temporal expression patterns: sub-cluster 1 (“rise-then-fall”) captures acute immune activation; sub-cluster 2 (“fall-then-rise”) reflects transient neural suppression followed by recovery; and sub-cluster 4 (“persistently elevated”) reveals a sustained immune-activated state that may drive chronic pathology.

To systematically uncover the dynamic patterns of gene expression during CVST progression, we performed hierarchical clustering on the 3502 DEGs identified across the three comparisons. Based on their expression trends across Sham, CVST 2d, and CVST 7d groups, three major expression patterns were identified (Supplementary Figure S3; Supplementary Table S7). Sub-cluster 1 (2328 genes) showed a “rise-then-fall” pattern (Figure 4A). Sub-cluster 2 (1108 genes) exhibited a “fall-then-rise” pattern (Figure 4B). Sub-cluster 4 (43 genes) displayed a “persistently elevated” pattern (Figure 4C).

Sub-cluster 1 (“rise-then-fall”) genes were enriched in immune system processes, defense response, and stress pathways (Supplementary Figure S4A). KEGG analysis revealed involvement in NF-κB, TNF, and NOD-like receptor signaling (Figure 4D), while Reactome highlighted innate immunity, neutrophil degranulation, and ECM organization (Supplementary Figure S4B). This pattern reflects acute immune inflammatory activation that resolves by the subacute phase.

Sub-cluster 2 (“fall-then-rise”) genes were associated with synaptic structure, ion channels, and neurotransmitter signaling (Figure 4E, Supplementary Figure S4C,D), suggesting transient neural suppression during acute injury followed by recovery-phase restoration.

Sub-cluster 4 (“persistently elevated”) genes showed sustained enrichment in immune regulation, complement activation, and microglial function (Supplementary Figure S4E). KEGG further implicated amino acid metabolism and PD-1/PD-L1 pathways (Figure 4F). Reactome highlighted persistent complement cascade and metabolic reprogramming (Supplementary Figure S4F). This suggests a chronic immune-activated state potentially linked to tissue repair and disease progression.

### 3.6. Quality Control of Proteomic Data

To investigate the protein-level regulatory mechanisms in CVST, we performed proteomic analysis on samples from Sham, CVST 2d, and CVST 7d groups (n = 3/group), generating 31.6 Gb of clean data (≥2.50 Gb/sample). Peptide coverage distribution showed most proteins (~2400) were identified by a single peptide, with peptide lengths concentrated between 7 and 20 amino acids (Supplementary Figure S5A,B). Identified proteins were predominantly <100 kDa; sequence coverage was generally low (20–40% for 30.74% of proteins) (Supplementary Figure S5C,D). In total, 123,646 peptides, 19,090 proteins, and 9706 protein groups were identified (Supplementary Figure S5E). The k-NN imputation method yielded a relative standard deviation of 0.354, substantially lower than that of zero imputation (1.044) (Supplementary Figure S5F). The intra-group coefficient of variation (CV) was < 10% across groups, with most CV values within 0–20% (Supplementary Figure S5G–J), confirming high reproducibility and data reliability for downstream analysis.

### 3.7. Differential Expression Analysis at the Proteomic Level

**Figure 5:**
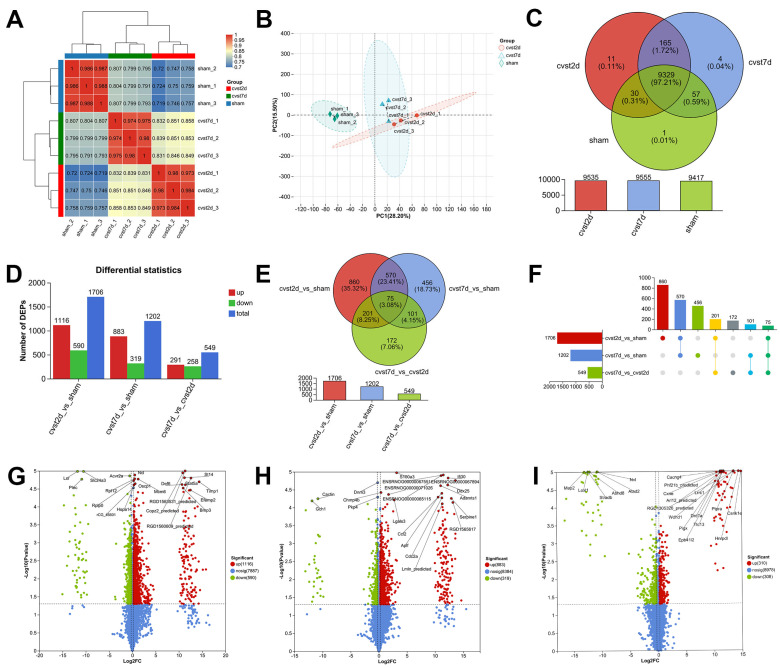
Differential proteomic analysis. (**A**) Inter-sample correlation heatmap. (**B**) Principal component analysis plot. (**C**) Venn diagram of identified proteins across the three groups. (**D**) Bar plot showing the number of differentially expressed proteins. (**E**,**F**) Venn diagram (**E**) and UpSet plot (**F**) of DEPs. (**G**–**I**) Volcano plots of differentially expressed proteins (corresponding to CVST 2d vs. Sham (**G**), CVST 7d vs. Sham (**H**), and CVST 7d vs. CVST 2d (**I**) comparisons, respectively. These data show that proteomic changes mirror the transcriptomic trends, with the most extensive protein alterations occurring in the acute phase, supporting a strong and early post-CVST proteome remodeling.

To systematically delineate the dynamic changes in the proteome during CVST progression, we conducted quantitative proteomic analysis on samples from Sham, CVST 2d, and CVST 7d groups. Inter-sample correlation (Pearson > 0.973) and PCA confirmed high intra-group reproducibility and clear separation of Sham, CVST 2d, and CVST 7d groups (Figure 5A,B). Protein identification yielded 9417 (Sham), 9535 (CVST 2d), and 9555 (CVST 7d) proteins, with 9329 shared (Figure 5C; Supplementary Table S8). DEP analysis identified 1706 DEPs (1116 up, 590 down) in CVST 2d vs. Sham; 1202 DEPs (883 up, 319 down) in CVST 7d vs. Sham; and 549 DEPs (291 up, 258 down) in CVST 7d vs. CVST 2d (Figure 5D; Supplementary Tables S9–S11). In total, 2435 non-redundant DEPs were identified, with distributions shown in Venn and UpSet plots (Figure 5E,F). Volcano plots display the top 20 most significantly altered proteins for each comparison (Figure 5G–I).

### 3.8. Temporal Clustering and Functional Enrichment Analysis of DEPs

**Figure 6:**
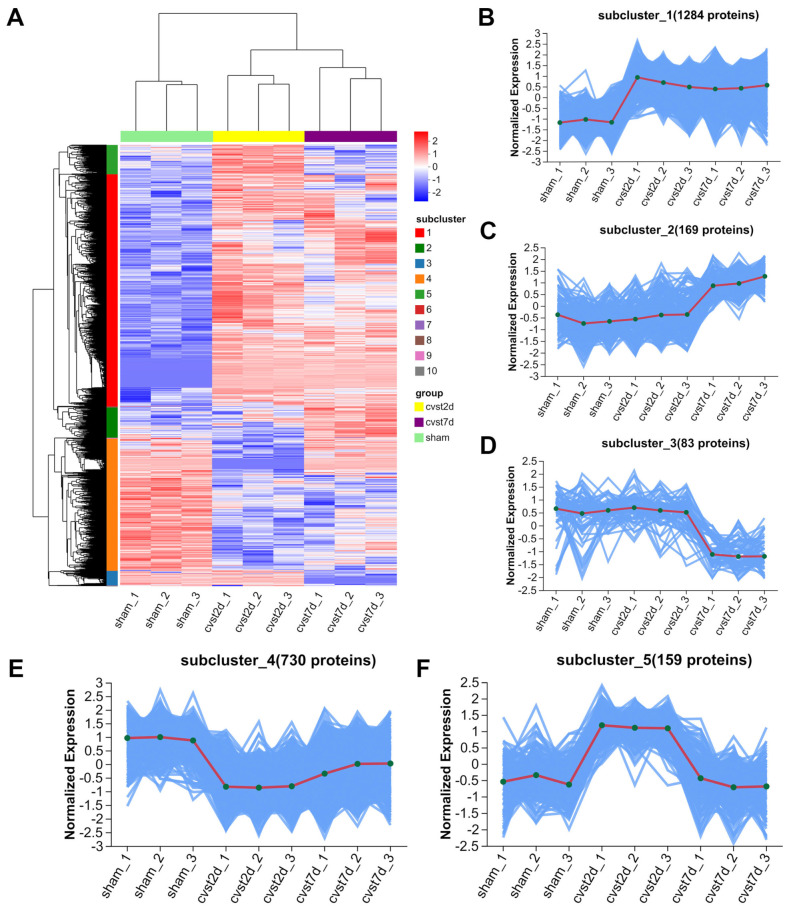
Clustering and expression trend analysis of differentially expressed proteins. (**A**) Clustering heatmap illustrating the division into ten initial sub-classes. (**B**–**F**) Mean expression trend plots for sub-classes 1–5, respectively. Lines represent individual gene expression trends; green dots show the mean expression of all genes per sample; the red line indicates the overall mean trend for each sub-cluster. These five temporal patterns reveal distinct protein regulation modules: persistent up-regulation (sub-class 1), delayed up-regulation (sub-class 2), delayed down-regulation (sub-class 3), acutely down regulated then partially recovered (sub-class 4), and transient acute up-regulation (sub-class 5). Such diversity highlights the complexity of post-transcriptional regulation during CVST progression.

To systematically resolve the dynamic patterns of protein expression during CVST progression, we performed hierarchical clustering on all 2435 DEPs. Based on their expression profiles across Sham, CVST 2d, and CVST 7d groups, five major expression sub-classes were defined (Figure 6A; Supplementary Table S12). Sub-class 1 (1284 proteins) increased at CVST 2d and remained elevated at CVST 7d (Figure 6B). Sub-class 2 (169) was stable in Sham/CVST 2d, up-regulated at CVST 7d (Figure 6C). Sub-class 3 (83) was high in Sham/CVST 2d, down-regulated at CVST 7d (Figure 6D). Sub-class 4 (730) was decreased at CVST 2d, partially recovered by CVST 7d (Figure 6E). Sub-class 5 (159) was up-regulated at CVST 2d, declined below baseline at CVST 7d (Figure 6F). These patterns provide a framework for investigating protein regulation across CVST stages.

**Figure 7:**
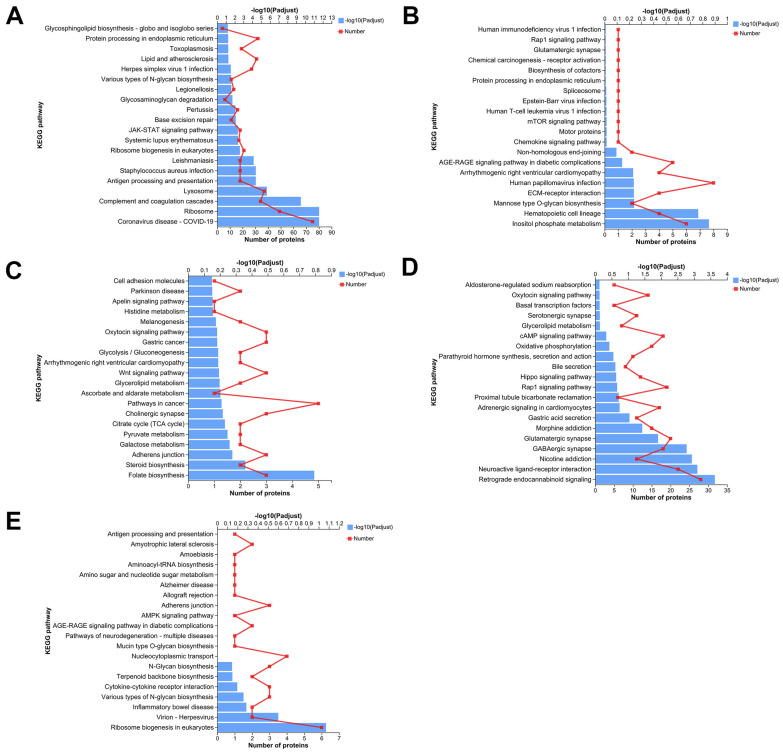
Functional enrichment analysis of five protein sub-classes. (**A**–**E**) Bar plots of KEGG pathway enrichment. Together, the five sub-classes correspond to sustained immunity, delayed repair, metabolic transition, neural recovery, and acute stress response, respectively.

GO, KEGG, and Reactome enrichment analyses revealed distinct functional signatures for each protein sub-class. Sub-class 1 (persistently up-regulated) proteins were enriched in immune processes, antigen presentation, and ribosome function (Supplementary Figure S6A). KEGG confirmed involvement in ribosome, antigen processing, and complement cascades (Figure 7A). Reactome highlighted translation initiation, innate immunity, and neutrophil degranulation (Supplementary Figure S6B). These findings indicate sustained immune activation and protein synthesis.

Sub-class 2 (delayed up-regulated) proteins were involved in cell chemotaxis, migration, and angiogenesis (Supplementary Figure S6C). KEGG showed enrichment in Rap1, mTOR, and chemokine signaling (Figure 7B). Reactome revealed roles in DNA repair and ECM organization (Supplementary Figure S6D). This suggests repair programs initiated in later stages.

Sub-class 3 (delayed down-regulated) proteins participated in differentiation, metabolism, and epigenetic regulation (Supplementary Figure S6E). KEGG implicated carbohydrate metabolism and Wnt signaling (Figure 7C). Reactome highlighted cellular senescence and DNA repair (Supplementary Figure S6F). Together, these results reflect metabolic reprogramming and transition to homeostasis.

Sub-class 4 (acutely down then partially recovered) proteins were enriched in synaptic structures, ion channels, and neuronal signaling (Figure 7D, Supplementary Figure S6G,H). This suggests acute neural suppression followed by gradual functional restoration.

Sub-class 5 (acutely transiently up-regulated) proteins were involved in ribosome biogenesis, rRNA processing, and stress response (Figure 7E, Supplementary Figure S6I,J). This indicates rapid activation of protein synthesis to meet acute stress demands.

### 3.9. Integrated Transcriptomic and Proteomic Analysis

**Figure 8:**
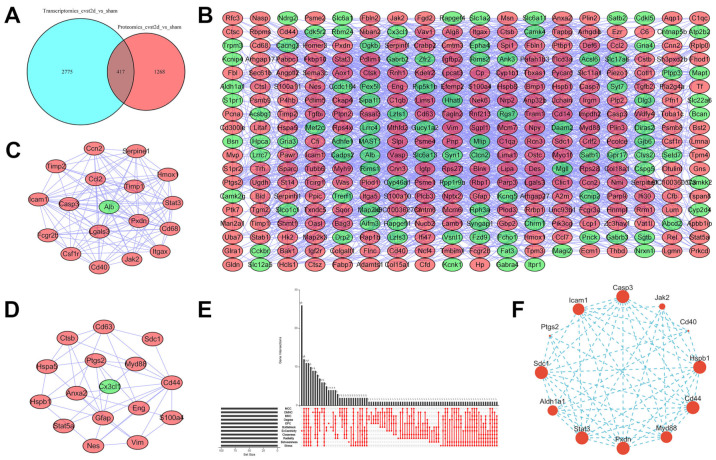
Integrated transcriptomic and proteomic analysis. (**A**) Venn diagram of the intersection of differentially expressed genes from transcriptomic and proteomic analyses. (**B**) Protein–protein interaction network (red, up-regulated; green, down-regulated). (**C**,**D**) Key functional modules identified by MCODE. (**E**) UpSet plot of core gene selection. (**F**) Co-expression network of the core genes. Twelve core genes were identified, showing exceptionally high co-expression (Spearman ≥ 0.8), indicating a tightly coordinated functional module that may serve as a synergistic biomarker panel for CVST.

To systematically identify core regulatory molecules in CVST pathogenesis, we performed an integrative analysis of transcriptomic and proteomic data. This analysis (CVST 2d vs. Sham) identified 417 common differentially expressed genes (Figure 8A). A PPI network of 336 genes was constructed using STRING (Figure 8B). MCODE identified 14 functional modules encompassing 99 genes (Supplementary Table S13). CytoHubba (11 algorithms) ranked gene importance. Intersection of the top 100 from each yielded 12 core genes: *Cd44*, *Cd40*, *Sdc1*, *Myd88*, *Icam1*, *Stat3*, *Jak2*, *Ptgs2*, *Aldh1a1*, *Hspb1*, *Pxdn*, and *Casp3* (Figure 8E; Supplementary Table S14). Eleven of these (except *Aldh1a1*) were located in MCODE Modules 1 and 3 (Figure 8C,D). Co-expression analysis showed exceptionally high correlation among these 12 genes (Spearman ≥ 0.8; Figure 8F), indicating a tightly coordinated functional module involved in immune inflammatory responses, cell adhesion, and stress signaling. This integrated pattern supports multi-target intervention strategies and suggests these genes as a synergistic biomarker panel for monitoring disease status or therapeutic response.

### 3.10. Multi-Omics Validation of Core Genes

**Figure 9:**
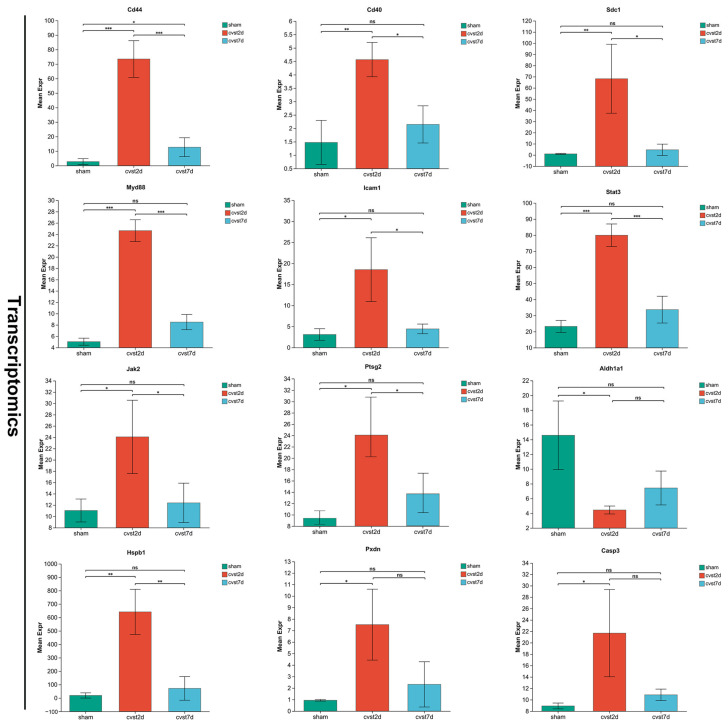
Transcriptomic validation of core gene expression. Expression differences of the 12 genes across the three groups at the transcriptomic level (one-way ANOVA). Significance: *** *p* < 0.001, ** *p* < 0.01, * *p* < 0.05; ns, not significant. These data confirm that 11 of the 12 core genes are significantly up-regulated at the acute phase (CVST 2d), whereas Aldh1a1 is down-regulated, indicating a coordinated transcriptional reprogramming in response to CVST.

**Figure 10:**
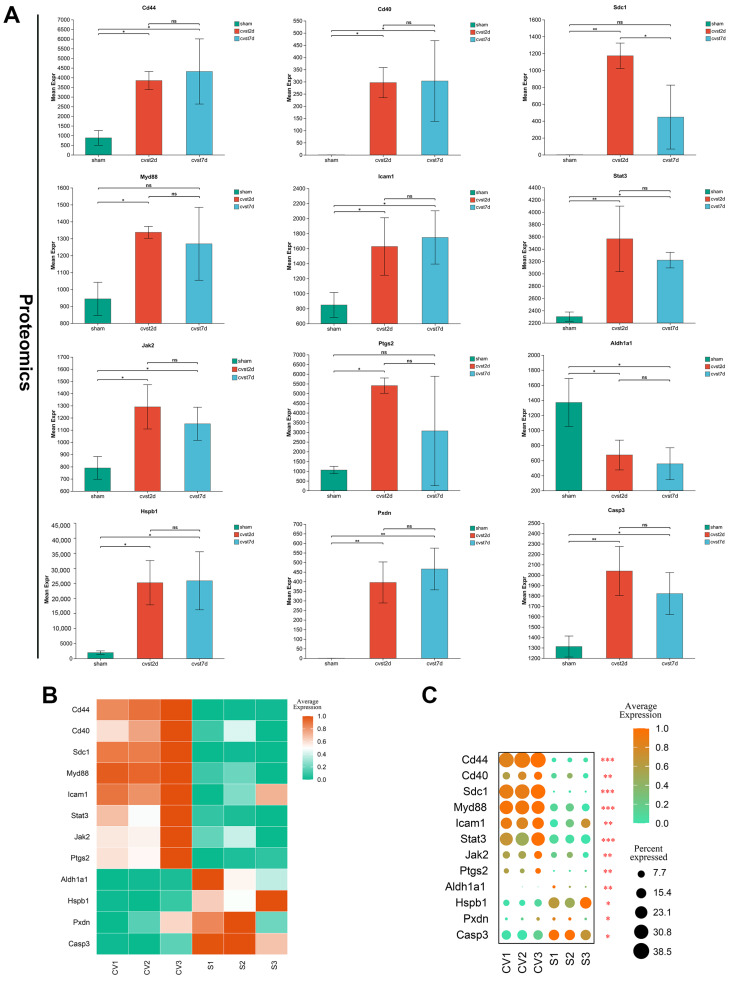
Proteomic and single-cell transcriptomic validation of core gene expression. (**A**) Expression differences of the 12 genes across the three groups at the proteomic level (one-way ANOVA). (**B**,**C**) Expression heatmap (**B**) and dot plot (**C**) of the 12 genes in single-cell transcriptomic data. Significance: *** *p* < 0.001, ** *p* < 0.01, * *p* < 0.05; ns, not significant. These data confirm that protein-level trends mirror transcriptomic changes, and single-cell analysis reveals cell-type-specific expression patterns. Notably, Hspb1, Pxdn, and Casp3 exhibit opposite trends at the single-cell level, underscoring the need for cellular-resolution interpretation.

To validate the expression patterns of the above 12 core genes during CVST progression, we performed cross-omics concordance analysis at the transcriptomic, proteomic, and single-cell transcriptomic levels. Transcriptomic analysis: one-way ANOVA showed significant differences across groups for all 12 genes (*p* < 0.05). At CVST 2d, 11 genes were up-regulated and *Aldh1a1* down-regulated versus Sham (Figure 9). Proteomic: protein-level trends were highly consistent with transcriptomic data (Figure 10A), indicating coordinated transcriptional and translational regulation.

Single cell: *Cd44*, *Cd40*, *Sdc1*, *Myd88*, *Icam1*, *Stat3*, *Jak2*, *Ptgs2*, and *Aldh1a1* (down) aligned with bulk data (Figure 10B,C). However, *Hspb1*, *Pxdn*, and *Casp3* showed opposite trends (down at single-cell level), reflecting post-CVST changes in cell composition and cell-specific transcriptional programs. This discrepancy likely reflects a marked expansion of microglial/macrophage populations after CVST, which elevates bulk tissue expression, while individual neurons suppress these stress-related genes to maintain survival—a cell-type-specific adaptive response. Thus, 8 of the 12 genes showed consistent up-regulation across all layers, 1 (Aldh1a1) showed consistent down-regulation, and the remaining 3 (Hspb1, Pxdn, Casp3) displayed opposite trends at the single-cell level, highlighting the importance of cell-type context in understanding their roles.

### 3.11. Cell-Type-Specific Expression Analysis of Core Genes

Single-cell transcriptomic analysis identified 12 major cell types in rat brain tissue: astrocytes, B cells, endothelial cells, fibroblasts, microglia, mononuclear phagocytes, mural cells, neurons, neutrophils, oligodendrocytes, plasma cells, and T cells. Expression differences of the 12 core genes between Sham and CVST groups across each cell type were further analyzed.

**Figure 11:**
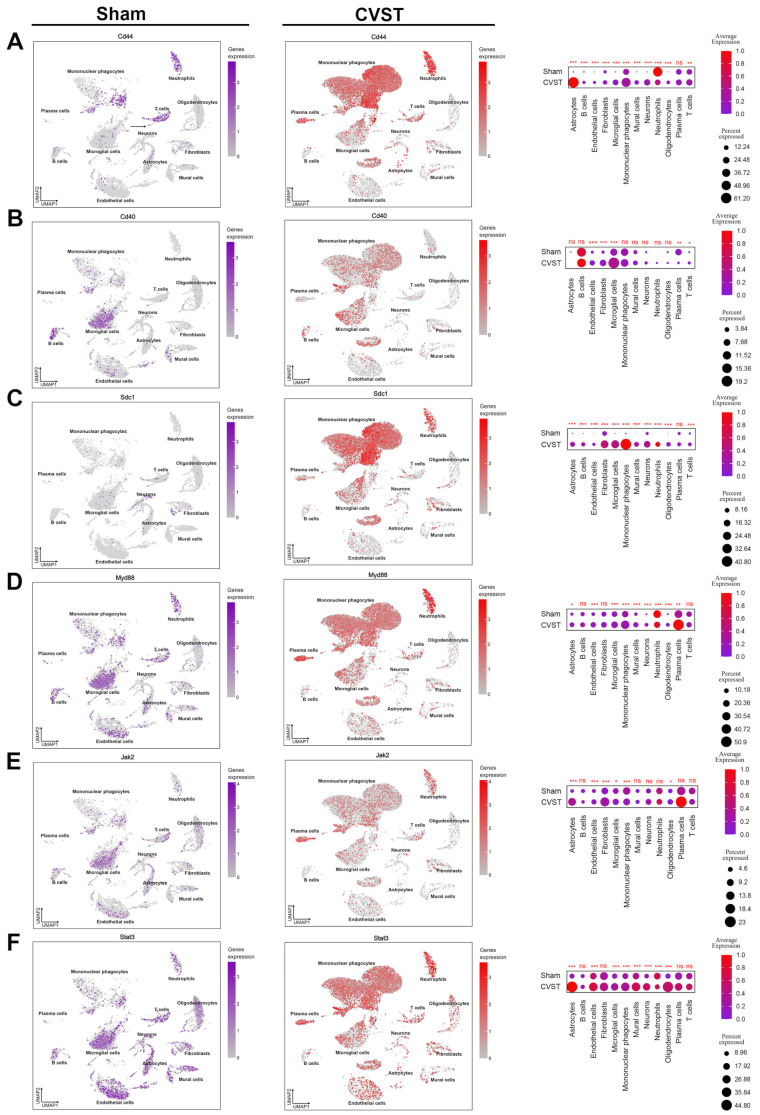
Cell-type-specific expression of six core genes. (**A**–**F**) show the expression levels of Cd44 (**A**), Cd40 (**B**), Sdc1 (**C**), Myd88 (**D**), Jak2 (**E**), and Stat3 (**F**) across 12 cell types in Sham and CVST groups. Significance: *** *p* < 0.001, ** *p* < 0.01, * *p* < 0.05; ns, not significant. These data reveal widespread but cell-type-specific dysregulation of core genes in CVST, with Jak2 and Stat3 exhibiting opposite trends in mononuclear phagocytes and neutrophils, highlighting the complexity of cell-specific immune responses.

*Cd44*, *Cd40*, *Sdc1*, *Myd88*, *Jak2*, and *Stat3* showed widespread cell-type-specific up-regulation in CVST (Figure 11A–F). *Cd44* was elevated in most cells but down in neutrophils (Figure 11A). *Cd40* increased in endothelial cells, fibroblasts, and microglia (Figure 11B). *Sdc1* rose in all cells except plasma cells (Figure 11C). *Myd88* was up-regulated in microglia and mononuclear phagocytes (Figure 11D). *Jak2* and *Stat3* were up-regulated in multiple types (astrocytes, endothelial cells, microglia) but showed opposite trends: *Jak2* down in mononuclear phagocytes (Figure 11E), *Stat3* down in neutrophils (Figure 11F).

The remaining six genes (*Icam1*, *Ptgs2*, *Aldh1a1*, *Hspb1*, *Pxdn*, *Casp3*) are shown in Supplementary Figure S7. *Hspb1* was up-regulated in astrocytes, fibroblasts, neurons but down in endothelial cells and microglia (Supplementary Figure S7D). *Casp3* was up-regulated in endothelial cells and fibroblasts yet down in myeloid immune cells (Supplementary Figure S7F). These opposing trends explain the bulk vs. single-cell discrepancy, reflecting heterogeneous transcriptional programs across cell types.

### 3.12. Drug Screening and Molecular Docking Targeting Core Proteins

**Figure 12:**
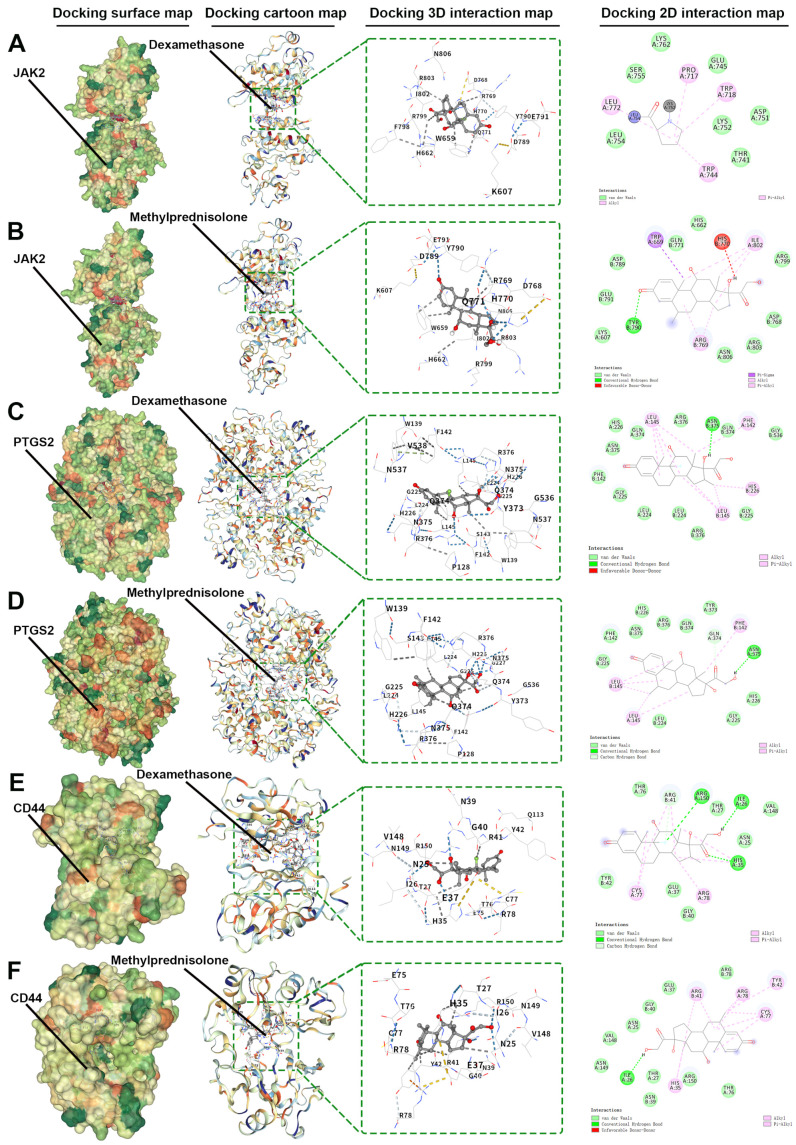
Representative molecular docking interaction diagrams depicting the binding modes of dexamethasone and methylprednisolone with JAK2, PTGS2, and CD44 proteins. (**A**) Dexamethasone with JAK2. (**B**) Methylprednisolone with JAK2. (**C**) Dexamethasone with PTGS2. (**D**) Methylprednisolone with PTGS2. (**E**) Dexamethasone with CD44. (**F**) Methylprednisolone with CD44. All complexes show binding energies < −8.2 kcal/mol, with methylprednisolone binding most strongly to PTGS2 (−11.2 kcal/mol), supporting the potential repurposing of glucocorticoids for CVST.

Using DGIdb and DSigDB, we identified eight candidates, including four glucocorticoids (dexamethasone, methylprednisolone, hydrocortisone, mometasone furoate), two immunomodulators (thalidomide, ibrutinib), one statin (simvastatin), and one JAK2 inhibitor (WP1066). All docked complexes showed binding energies <−8.2 kcal/mol (Table 1). Dexamethasone, methylprednisolone, and several others bound JAK2 (−9.7 to −8.4 kcal/mol). For PTGS2, methylprednisolone showed the strongest affinity (−11.2 kcal/mol). Dexamethasone and methylprednisolone also bound CD44 (−9.0 to −8.5 kcal/mol). Ibrutinib bound MYD88 (−8.2 kcal/mol), and simvastatin bound ICAM1 (−8.5 kcal/mol). Key interactions are visualized in Figure 12.

### 3.13. Molecular Dynamics Simulation of Methylprednisolone and CD44

**Figure 13:**
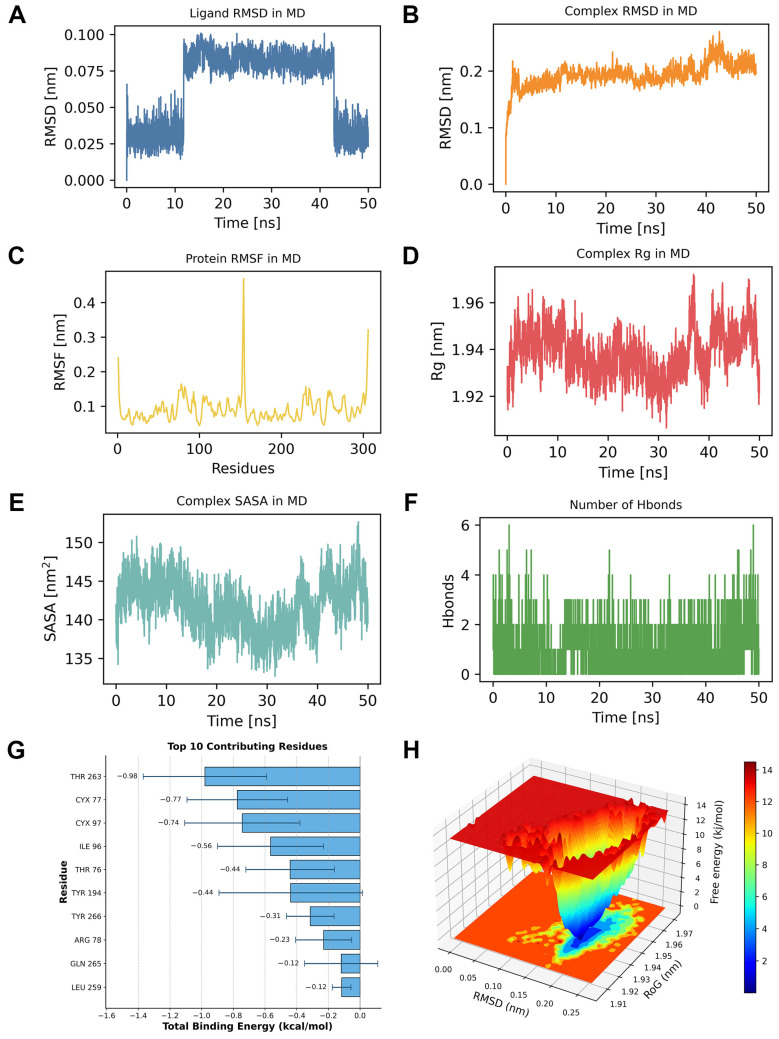
Molecular dynamics simulation results for methylprednisolone and CD44. (**A**) RMSD of methylprednisolone. (**B**) RMSD of the complex. (**C**) RMSF of CD44. (**D**) Rg of the complex. (**E**) SASA of the complex. (**F**) Number of hydrogen bonds. (**G**) Binding energy contribution of key residues. (**H**) Free energy landscape. Rg, radius of gyration; RMSD, root-mean-square deviation; RMSF, root-mean-square fluctuation; SASA, solvent-accessible surface area. Together, these data confirm that methylprednisolone binds CD44 tightly and stably (RMSD 0.18–0.22 nm, ΔG = −13.85 kcal/mol), driven mainly by van der Waals and electrostatic interactions, with cooperative contributions from several polar residues.

To assess the stability of the binding between methylprednisolone and CD44, we performed a 50 ns molecular dynamics simulation. For methylprednisolone, the RMSD remained low over the first 10 ns. It then increased around 10 ns and remained stable at a higher level between 10 and 40 ns. After 40 ns, it decreased again (Figure 13A). This up-down-up pattern suggests a reversible change in ligand binding mode. Nevertheless, the overall RMSD shift was modest. This indicates that the different binding states exhibited limited structural differences. Importantly, the ligand never left the binding pocket.

For the complex, its RMSD rose quickly at the start but soon stabilized within a range of 0.18–0.22 nm (Figure 13B). This indicates that the complex remained structurally sound, and drug binding did not cause any major backbone rearrangements.

The RMSF values of CD44 were generally low across the board. Only a few residues showed noticeable peaks, with the highest around 0.45 nm (Figure 13C). This suggests that the protein core is fairly rigid. The few flexible regions (likely loops or parts of the binding pocket entrance) probably provide the ligand with conformational adaptability. The Rg remained within a narrow range of 1.92–1.96 nm (Figure 13D). The SASA followed a similarly steady pattern (Figure 13E). Both observations indicate good structural integrity of the complex.

Hydrogen bond counts varied considerably, ranging from 0 to 6 (Figure 13F). Clear fluctuations occurred around the times the ligand switched conformations (approximately 10 ns and 40 ns). This suggests the hydrogen bond network underwent rearrangement. The relatively stable hydrogen bond count during the middle phase indicates that a stable interaction pattern was established during that period.

MM-GBSA calculations yielded a binding free energy of −13.85 ± 3.42 kcal/mol for the methylprednisolone–CD44 complex (Supplementary Table S15). The negative value confirms strong binding affinity. Decomposition of the energy components revealed that van der Waals (−21.67 ± 6.07 kcal/mol) and electrostatics (−30.75 ± 8.87 kcal/mol) contributions were the main drivers.

Analysis of individual residues showed that the energy contributions were fairly evenly distributed, with no single residue dominating. This indicates a cooperative binding mechanism involving multiple residues. Among them, several polar residues, especially THR 263, THR 76, and GLN 265, made notable contributions (Figure 13G).

The free energy landscape showed a single sharp energy well (Figure 13H), with the lowest-energy region located at moderate RMSD and a relatively compact Rg. This indicates that the complex remains globally stable while still allowing some local flexibility.

The stable binding of methylprednisolone to CD44 (RMSD < 0.22 nm, binding energy −13.85 kcal/mol) suggests that glucocorticoids could physically interact with this target at a clinically relevant affinity. This strengthens the rationale for repurposing dexamethasone or methylprednisolone in CVST patients, potentially reducing thrombosis-associated neuroinflammation.

Collectively, these results demonstrate that methylprednisolone binds tightly to CD44, with van der Waals and electrostatic forces serving as the primary drivers and multiple residues contributing cooperatively to the stability of the complex.

### 3.14. Post-Transcriptional and Transcriptional Regulatory Network Analysis of Core Genes

A ceRNA network was constructed for core genes. SDC1 was predicted to interact with miRNAs (e.g., hsa-miR-665, hsa-miR-9-5p) and form regulatory axes with lncRNAs (RP5-894D12.5, SNHG14, RASSF8-AS1) (Supplementary Figure S8A). Pxdn also interacted with hsa-miR-9-5p, while Stat3 and Casp3 participated in similar networks (Supplementary Figure S8B). LncRNAs (AC091153.4, CTC-457E21.1, RP1-182D15.2) were predicted to compete with Ptgs2, Jak2, Cd44, and Icam1 for shared miRNA binding (Supplementary Figure S8C–E).

Upstream TFs were predicted using TRRUST (Table 2). Examples: CD44 activated by SP1/TWIST1, repressed by HDAC1/SMARCA4; ICAM1 activated by NFKB1/RELA/STAT1, repressed by NFKBIA/PPARG; PTGS2 activated by CREB1/NFKB1/STATs, repressed by PPARG/USF1; STAT3 activated by HIC1, repressed by TP53/ZNF382; JAK2 activated by STAT1/STAT3.

### 3.15. Expression Profiles of Potential Edema-Related Therapeutic Targets in CVST

**Figure 14:**
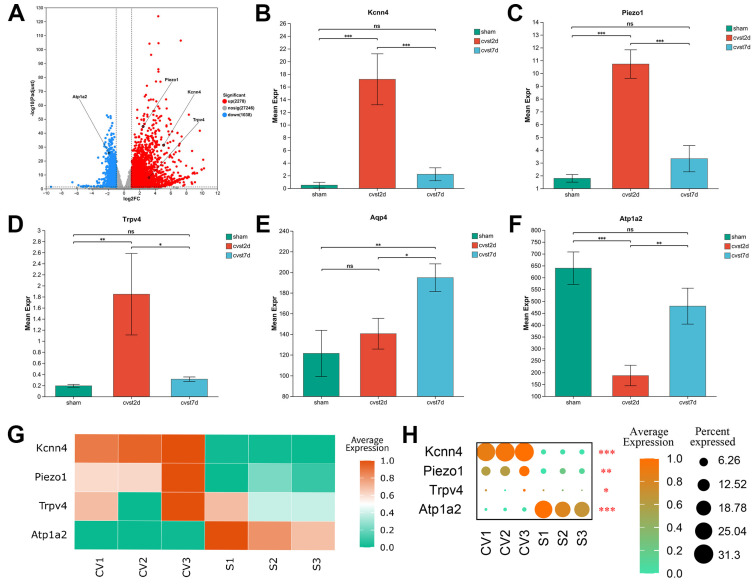
Expression analysis of potential edema-related targets. (**A**) Volcano plot of differentially expressed genes (Sham vs. CVST 2d). (**B**–**F**) Temporal expression differences of Kcnn4, Piezo1, Trpv4, Aqp4, and Atp1a2 at the transcriptomic level (one-way ANOVA). (**G**,**H**) Single-cell transcriptomic expression heatmap and dot plot. Significance: *** *p* < 0.001, ** *p* < 0.01, * *p* < 0.05; ns, not significant. Ion channels (Kcnn4, Piezo1, Trpv4) are acutely up-regulated, while Aqp4 increases later, indicating time-dependent roles in CVST-induced edema.

Expression of edema-associated genes showed distinct temporal patterns (Figure 14A–F): Kcnn4, Piezo1, and Trpv4 were up-regulated at CVST 2d and declined by CVST 7d (Figure 14B–D). Atp1a2 was down-regulated at CVST 2d and partially recovered at CVST 7d (Figure 13F). Aqp4 showed no change at CVST 2d but was up-regulated at CVST 7d (Figure 14E). Single-cell analysis confirmed these trends for Kcnn4, Piezo1, Trpv4, and Atp1a2 (Figure 14G,H), indicating dynamic regulation of ion/water homeostasis genes in CVST-associated edema.

## 4. Discussion

CVST is characterized by impaired cerebral blood flow due to venous obstruction, with headache as the primary manifestation [28]. Misdiagnosis rates are high, and patients often develop severe brain damage and intracranial hypertension [4,29]. Molecular mechanisms underlying CVST-induced brain injury remain poorly understood. Using a modified rat CVST model with multi-omics profiling, we systematically identified key molecular targets and dysregulated pathways associated with post-CVST cerebral pathology.

Current CVST models are time-consuming and poorly reproducible [16,17]. Our modified model successfully induced SSS blood flow interruption and venous infarction. Functional enrichment revealed dynamic molecular changes across acute to subacute phases, suggesting evolving pathological mechanisms. Integrated analysis identified 12 core genes—*Cd44*, *Cd40*, *Sdc1*, *Myd88*, *Icam1*, *Stat3*, *Jak2*, *Ptgs2*, *Aldh1a1*, *Hspb1*, *Pxdn*, and *Casp3*—that may collectively mediate CVST pathogenesis. Single-cell sequencing resolved cell-type-specific expression patterns and validated bulk data reliability. Additionally, drug docking, molecular dynamics, and network analysis provided therapeutic insights.

CD44, a transmembrane glycoprotein, plays diverse roles in inflammation and neurorepair [30,31]. In cerebral ischemia, CD44 up-regulation in microglia amplifies pro-inflammatory signals via SRGN interaction [32], while hyaluronic acid-CD44 interaction compromises endothelial barrier integrity. Paradoxically, CD44 interaction with galectin-9 promotes oligodendrocyte differentiation and remyelination, aiding recovery [33]. In our CVST model, CD44 was up-regulated in multiple cell types across acute and subacute phases, suggesting stage- and cell-specific modulation may offer therapeutic benefit. Single-cell data further showed that Cd44 was up-regulated mainly in microglia, astrocytes, and infiltrating immune cells but down-regulated in neutrophils. This suggests that CD44’s pro-inflammatory role in CVST may be driven by microglial and astrocytic activation [32,34]. The down-regulation of Cd44 in neutrophils could potentially limit excessive neutrophil infiltration, which might represent a protective feedback mechanism [34].

CD40 (TNFRSF5), expressed on immune and non-immune cells, initiates and sustains inflammation [35,36]. CD40L promotes microthrombosis by inducing endothelial adhesion molecules [37]. Inhibiting CD40/CD40L signaling reduces neuroinflammation and infarct volume in stroke models [38]. In CVST patients, CD40-CD40L up-regulation correlates with pro-thrombotic markers [39]. Our data showed elevated CD40 in endothelial cells, microglia, and fibroblasts post-CVST, implicating it in barrier disruption and inflammation.

The JAK2-STAT3 pathway mediates cytokine signaling and neuroinflammation [40]. Its effects are context-dependent: inhibition improves blood–brain barrier (BBB) integrity acutely [41], while activation promotes repair [42]. The JAK2-V617F mutation worsens CVST prognosis [43]. Our data showed sustained JAK2-STAT3 activation post-CVST. Single-cell analysis revealed that Jak2 and Stat3 were up-regulated in astrocytes, endothelial cells, and microglia, but Jak2 was suppressed in mononuclear phagocytes and Stat3 in neutrophils. This cell-type-specific divergence implies that JAK2-STAT3 signaling promotes inflammation in glial and endothelial compartments [44], while its down-regulation in phagocytes and neutrophils may represent a compensatory brake on systemic inflammation [45]. These findings suggest dual roles of the pathway, warranting spatiotemporally targeted modulation.

ICAM-1 mediates leukocyte–endothelial adhesion and BBB dysfunction [46,47]. Syndecan-1 shedding exacerbates vascular permeability and inflammation [48,49]. MyD88 drives pro-inflammatory signaling via Toll-like receptor 4 [50]. Single-cell data showed that Icam1 was most highly expressed in endothelial cells and fibroblasts, supporting its role in endothelial–leukocyte adhesion and perivascular fibroblast activation. Sdc1 was broadly expressed across multiple cell types, while Myd88 was enriched in microglia and mononuclear phagocytes. These cell-type-specific patterns suggest distinct contributions: ICAM-1 in vascular and stromal cells, Syndecan-1 in diverse cell populations, and MyD88 in immune cells. Our multi-omics data implicate these molecules in CVST, but further functional studies are needed to dissect their cell-specific roles.

Notably, Hspb1, Pxdn, and Casp3 showed opposite trends between bulk and single-cell data. This reflects microglial/macrophage expansion driving bulk up-regulation, while neuronal down-regulation suggests cell-specific responses. Such heterogeneity underscores the need for single-cell resolution in disease mechanism studies.

Glucocorticoids (dexamethasone, methylprednisolone) are widely used anti-inflammatory agents [51]. Duan et al. reported that dexamethasone reduces thrombus burden and improves outcomes in CVST patients [52,53]. Consistent with these findings, our docking analysis revealed strong binding of glucocorticoids to JAK2, PTGS2, and CD44, suggesting these may be direct molecular targets. To further assess binding stability, molecular dynamics simulations indicated that methylprednisolone forms a stable complex with CD44. Nevertheless, the clinical efficacy of glucocorticoids in CVST awaits validation in prospective multicenter trials.

Cerebral edema is a major cause of poor prognosis after CVST [29]. Its pathogenesis involves Na^+^/K^+^ ATPase dysfunction (reflected by reduced Atp1a2 expression) [54], aquaporin dysregulation [55], glymphatic impairment [56], and ion channel activity [57]. *AQP4*, essential for water homeostasis and glymphatic function, shows polarized expression at perivascular astrocyte end-feet [56,58]. *TRPV4*, enriched in astrocytes and endothelial cells [59], influences glymphatic flow and edema resolution [60]. *Piezo1*, expressed in vascular and lymphatic cells [61,62], regulates BBB integrity and meningeal drainage [63]. In our CVST model, *Trpv4*, *Piezo1*, and *Kcnn4* were up-regulated at 2 days, while Aqp4 increased only at 7 days. This temporal pattern aligns with AQP4’s dual role in edema formation and clearance [60,64]. These findings implicate ion and water channels in CVST-associated edema, though precise mechanisms require further study.

Several limitations of this study should be acknowledged. First, while our integrated multi-omics analysis identified 12 core genes, functional validation (e.g., gene knockdown/knockout or pharmacological inhibition) in the CVST model is required to establish causality. Second, the candidate compounds were evaluated only in silico; the assumption that computational binding energies directly correlate with in vivo efficacy needs experimental verification (e.g., animal efficacy studies). Third, our rat CVST model, although widely used, may not fully recapitulate all aspects of human CVST, particularly differences in thrombus resolution, immune response kinetics, and the presence of comorbid conditions in patients. Fourth, for proteomic differential expression analysis, we applied a nominal *p* < 0.05 threshold combined with a fold-change cutoff (>1.2 or <0.83), rather than FDR correction. This was a deliberate choice to balance sensitivity against the inherently lower dynamic range of proteomic data; however, we acknowledge that this approach may increase the risk of false positives. Future studies with orthogonal validation (e.g., targeted proteomics or Western blotting) will be needed to confirm the identified DEPs. Fifth, the sample size for omics analyses (n = 3 per group) is standard for exploratory multi-omics studies and is sufficient to identify large-effect molecular changes. However, we acknowledge that it may limit statistical power for detecting smaller effect sizes, and future studies with larger cohorts will be needed to validate subtle but potentially relevant changes. Sixth, the single-cell dataset was obtained from only two time points (Sham and 2d), and longitudinal changes in cell-type composition beyond 7 days were not captured. Seventh, we did not include a naive (untouched) control group. However, the Sham-operated group controlled for surgical stress and anesthesia, as it underwent the same procedures (craniotomy, clip placement, wound closure) as the CVST groups. Additionally, pentobarbital anesthesia may influence inflammatory gene expression, but because all groups received the same regimen, any such effect is likely balanced, and the differences between CVST and Sham groups are unlikely to be solely attributable to anesthesia. Future studies should include naive controls and consider using volatile anesthetics such as isoflurane. Finally, we did not perform in vitro assays (e.g., endothelial permeability or microglial cytokine release) to directly test the functional consequences of target modulation. Future studies addressing these limitations will strengthen the translational potential of our findings.

## 5. Conclusions

In summary, by applying integrated multi-omics profiling to a modified rat model, this study systematically mapped the molecular landscape of CVST-induced brain injury. A number of candidate targets and signaling pathways emerged from our analysis, along with several compounds of potential therapeutic interest. Collectively, these results provide a basis for further investigation into the mechanisms underlying CVST and for the design of novel treatment approaches.

## Figures and Tables

**Table 1:** Molecular docking binding energies for five target proteins with eight compounds.

Target	PDB ID	Drug	PubChem ID	Binding Energy
CD44	4PZ4	Dexamethasone	5743	−8.6 kcal/mol
CD44	4PZ4	Methylprednisolone	6741	−9.0 kcal/mol
CD44	4PZ4	Hydrocortisone	5754	−8.6 kcal/mol
CD44	4PZ4	Mometasone furoate	441336	−8.5 kcal/mol
ICAM1	1P53	Simvastatin	54454	−8.5 kcal/mol
JAK2	7F7W	Dexamethasone	5743	−9.1 kcal/mol
JAK2	7F7W	Methylprednisolone	6741	−9.3 kcal/mol
JAK2	7F7W	Thalidomide	5426	−9.7 kcal/mol
JAK2	7F7W	WP1066	11210478	−8.4 kcal/mol
JAK2	7F7W	Hydrocortisone	5754	−8.6 kcal/mol
JAK2	7F7W	Simvastatin	54454	−9.0 kcal/mol
MYD88	4DOM	Ibrutinib	24821094	−8.2 kcal/mol
PTGS2	5IKT	Dexamethasone	5743	−9.8 kcal/mol
PTGS2	5IKT	Methylprednisolone	6741	−11.2 kcal/mol
PTGS2	5IKT	Thalidomide	5426	−9.9 kcal/mol
PTGS2	5IKT	Hydrocortisone	5754	−9.5 kcal/mol
PTGS2	5IKT	Simvastatin	54454	−9.1 kcal/mol

**Table 2:** Predicted upstream transcription factors of core genes.

Gene	TF	Role
CD44	HMGA1, MYCN, SNAI2, SP1, TWIST1	Activation
	HDAC1, IKBKB, SMARCA1, SMARCA4, SMARCB1	Repression
	CTNNB1, TCF4	Unknown
CD40	IRF1, NFKB1, NR3C2,RELA, SPI1, SPIB, STAT1	Activation
	NFKBIA, TRERF1	Repression
	RELB, STAT6, TRAF6, XRCC5, XRCC6	Unknown
SDC1	NFKB1	Activation
MYD88	STAT3	Unknown
ICAM1	ETS2, ETV5, HDAC1, NFATC1, NFKB1, RARA, RARG, RELA, STAT1, TWIST1, TWIST2	Activation
	ERG, IFI16, MYB, NFKBIA, PPARG, SIRT1, CEBPA	Repression
	REL, SP1, STAT3	Unknown
STAT3	HIC1	Activation
	KAT5, TP53, ZNF382	Repression
	BCL6, BRCA1, CEBPA, HDAC1, HIF1A, PIAS3, RELA, SPI1, STAT1, TCF7L2	Unknown
JAK2	STAT1, STAT3	Activation
	BRCA1, ESR1	Unknown
PTGS2	CDX1, CEBPB, CREB1, DR1, EP300, ETV4, FOS, HDAC1, HDAC4, HMGA1, JUN, JUNB, JUND, NFKB1, NR0B2, PPARA, RELA, SP1, STAT1, STAT2, STAT3, STAT6	Activation
	APC, AR, CDX2, EGR1, ING4, PGR, PPARG, SETBP1, USF1	Repression
	ATF2, ATF4, CEBPD, CREBBP, CTNNB1, EGR2, ELF3, ENO1, NFIL3,TCF7L2, TFAP2A, USF2	Unknown
ALDH1A1	EZH2	Repression
	TLX1	Unknown
HSPB1	ESR1, STAT3	Activation
CASP3	ING1, ING4, PML, RUNX3, SIM2	Activation
	NFKB1, PHF10, RELA, SP1, TP53	Unknown

## Data Availability

The results of this study are available in the article and Supplementary Materials. The raw sequencing data (transcriptomic, proteomic, and single-cell RNA-seq) generated in this study are available from the corresponding author on reasonable request.

## Supplementary Materials

The following supporting information can be downloaded at: https://www.mdpi.com/article/10.3390/biomedicines14071594/s1. Supplementary Materials and Methods [26,65,66,67]. Table S1: Summary of sequencing quality control metrics; Table S2: Summary of sequence alignment results; Table S3: Summary of expressed genes identified in the sham, CVST 2d, and CVST 7d group; Table S4: Summary of DEGs in CVST 2d vs. Sham comparison; Table S5: Summary of differentially expressed genes in CVST 7d vs. Sham comparison; Table S6: Summary of differentially expressed genes in CVST 7d vs. CVST 2d comparison; Table S7: Summary of gene counts across 10 sub-clusters; Table S8: Summary of proteins identified in the sham, CVST 2d, and CVST 7d group; Table S9: Summary of differentially expressed proteins in CVST 2d vs. Sham comparison; Table S10: Summary of differentially expressed proteins in CVST 7d vs. Sham comparison; Table S11: Summary of differentially expressed proteins in CVST 7d vs. CVST 2d comparison; Table S12: Summary of protein counts across 10 sub-clusters; Table S13: Summary of MCODE analysis results; Table S14: Scoring results of 11 algorithms in the cytoHubba analysis; Table S15: Binding free energies and energy components predicted by MM/GBSA (kcal/mol). Figure S1: Quality assessment and expression distribution of transcriptome sequencing data; Figure S2: Functional annotation and enrichment analysis of DEGs; Figure S3: Heatmap of 10 sub-clusters; Figure S4: Clustering and functional enrichment analysis of DEGs; Figure S5: Quality control of proteomic data; Figure S6: Functional enrichment analysis of five protein sub-classes; Figure S7: Cell-type-specific expression of six core genes; Figure S8: ceRNA regulatory network of core genes. Orange nodes: core genes; cyan nodes: miRNAs; blue nodes: lncRNAs.

## Abbreviations

ARRIVE, Animal Research: Reporting of In Vivo Experiments; BBB, blood-brain barrier; BH, Benjamini–Hochberg; CVST, cerebral venous sinus thrombosis; ceRNA, competing endogenous RNA; DIA, data-independent acquisition; DEGs, differentially expressed genes; DEPs, differentially expressed proteins; DSigDB, drug signatures database; DGIdb, drug–gene interaction database; FeCl_3_, ferric chloride; GO, Gene Ontology; i.p., intraperitoneal injection; KEGG, Kyoto Encyclopedia Of Genes and Genomes; lncRNAs, long non-coding RNA; ANOVA, one-way analysis of variance; PCA, principal component analysis; PPI, protein–protein interaction; Rg, radius of gyration; RMSD, root-mean-square deviation; RMSF, root-mean-square fluctuation; ROI, region of interest; SASA, solvent-accessible surface area; SSS, superior sagittal sinus; TFs, transcription factors; TPM, transcripts per million; TTC, 2,3,5-Triphenyltetrazolium Chloride.
