# Supplementary material for: Integrated Multi-Omics Identifies Core Molecular Targets in Cerebral Venous Sinus Thrombosis-Induced Brain Injury
Source: Biomedicines. 2026 Jul 16;14(7):1594. doi: 10.3390/biomedicines14071594 (PMC13406610; doi:10.3390/biomedicines14071594)
Supplement: Supplementary file 1 [file biomedicines-14-01594-s001.zip › Supplementary Figure.pdf]

Supplementary Figures

Supplementary Figure S1. Quality assessment and expression distribution of transcriptome sequencing data.

Supplementary Figure S2. Functional annotation **a**nd enrichment analysis of DEGs.

Supplementary Figure S3. Heatmap of 10 sub-clusters.

Supplementary Figure S4. Clustering and functional enrichment analysis of DEGs.

Supplementary Figure S5. Quality control of proteomic data.

Supplementary Figure S6. Functional enrichment analysis of five protein sub-classes.

Supplementary Figure S7. Cell-type-specific expression of six core genes.

Supplementary Figure S8. ceRNA regulatory network of core genes. Orange nodes: core genes; cyan nodes: miRNAs; blue nodes: lncRNAs.

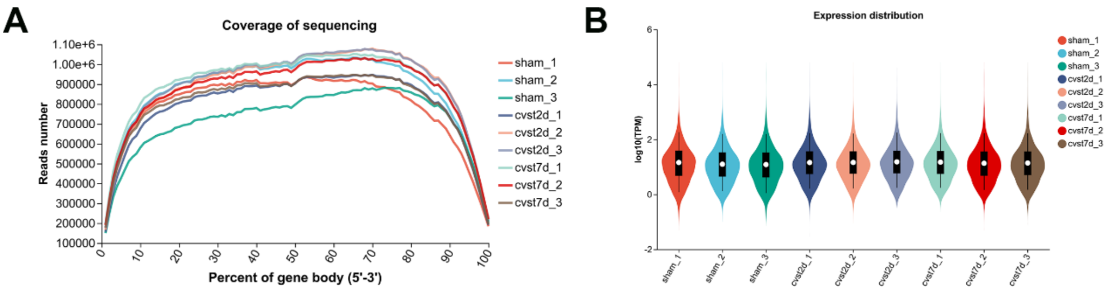

Supplementary Figure S1. Quality assessment and expression distribution of transcriptome sequencing data. (A) Sequencing coverage distribution curve. The x-axis indicates the relative position along genes (5′→3′), and the y-axis represents the total number of reads covering each position. (B) Violin plots depicting expression distribution across samples. The x-axis shows sample names, and the y-axis represents $\log_{10}$(expression level). Distinct colors denote different samples, with the broadened regions of each violin reflecting the concentration of gene expression values.

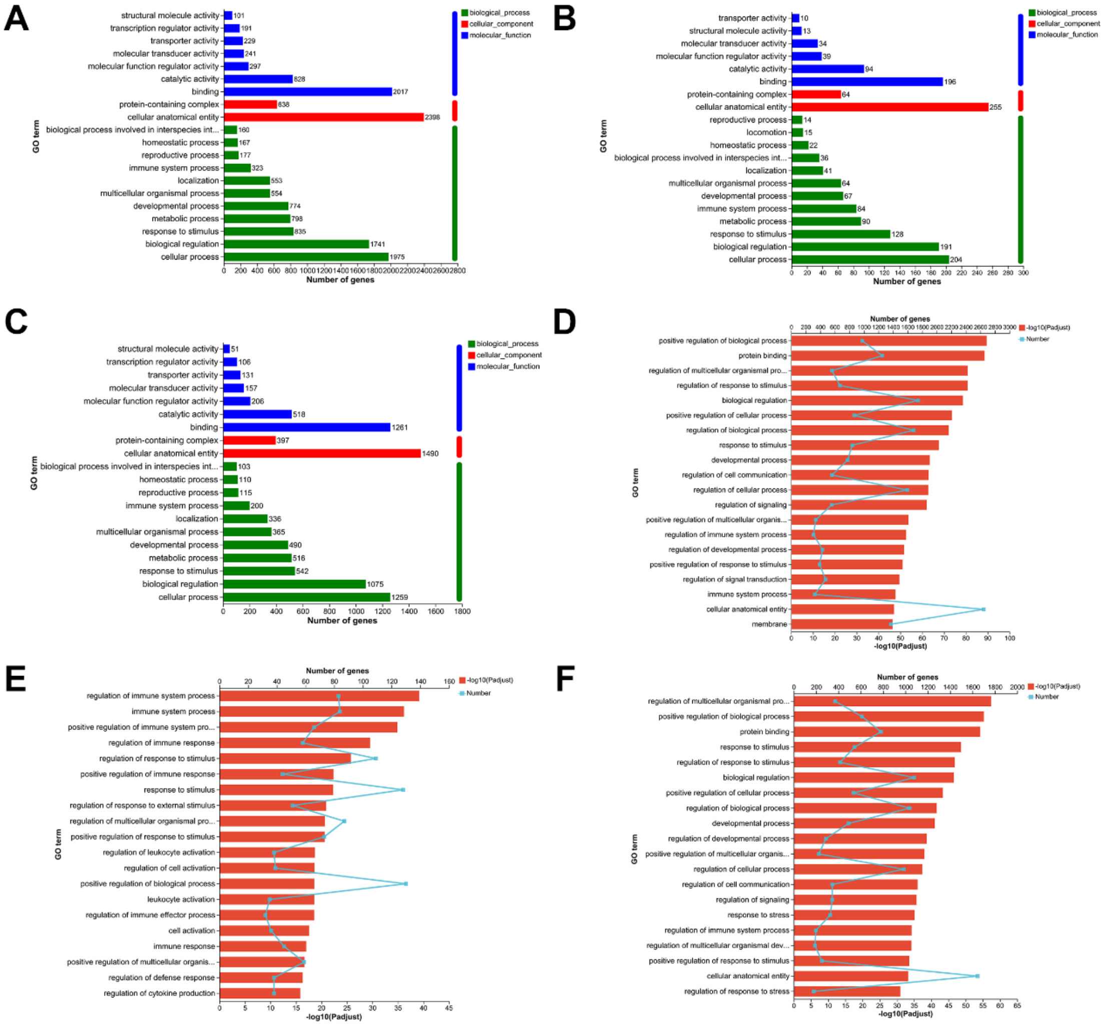

Supplementary Figure S2. Functional annotation and enrichment analysis of DEGs. (A–C) Bar plots illustrating the distribution of DEGs across the three GO categories—biological process, cellular component, and molecular function—for the three comparisons: CVST 2d vs

Sham (A), CVST 7d vs Sham (B), and CVST 7d vs CVST 2d (C). (D–F) Bar plots of GO enrichment analysis.

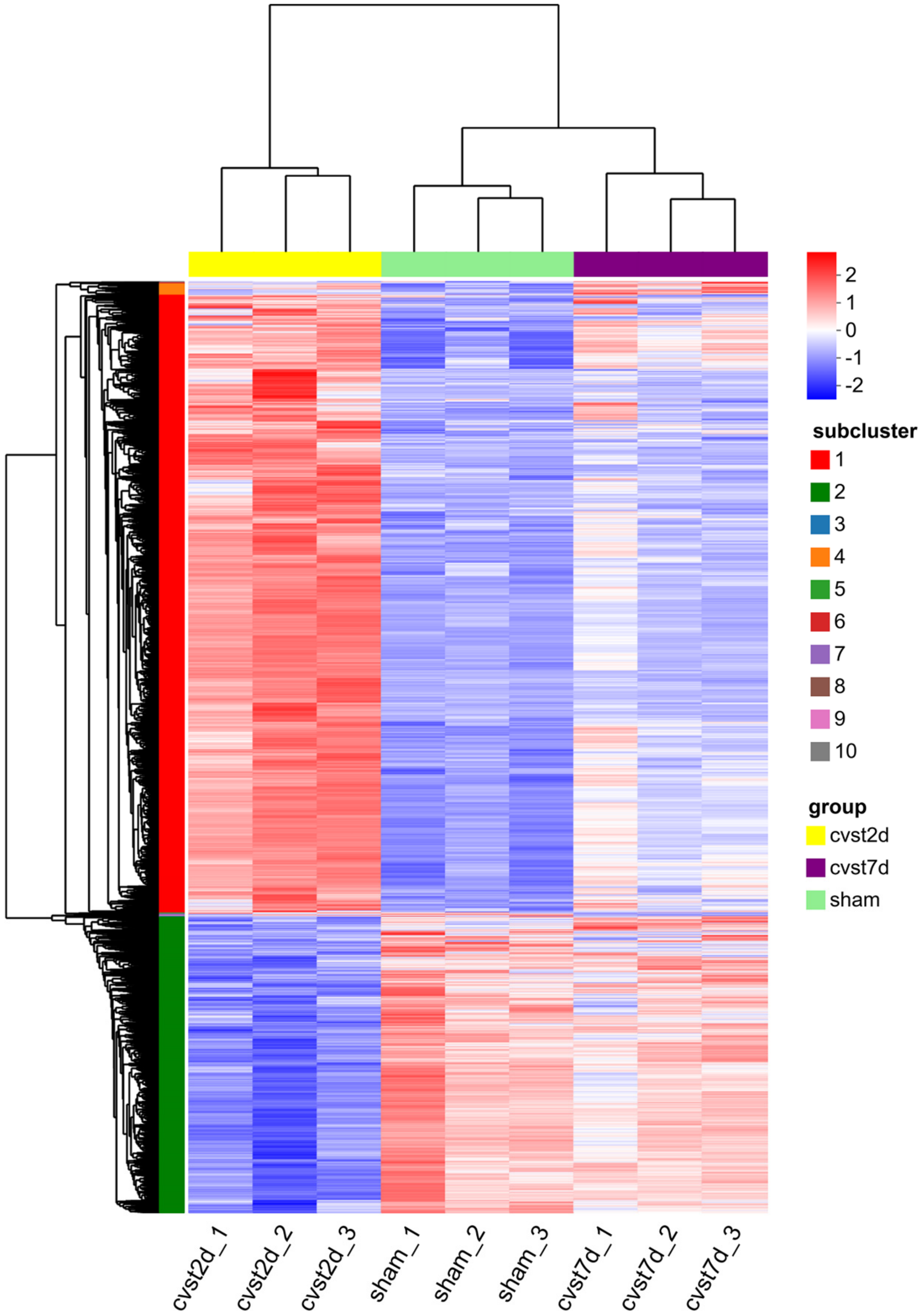

Supplementary Figure S3. Heatmap of 10 sub-clusters.

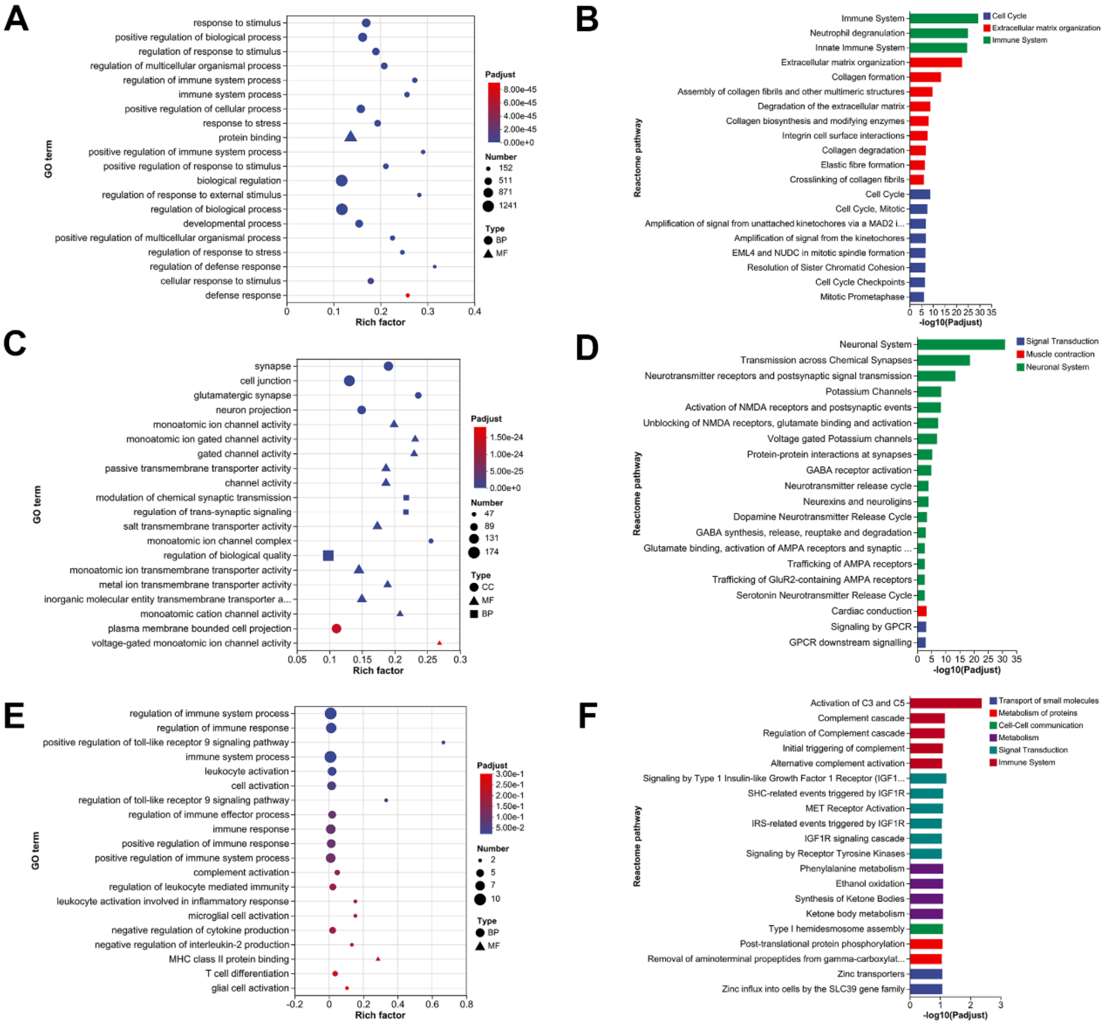

Supplementary Figure S4. Clustering and functional enrichment analysis of DEGs. (A, C, E) Bubble plots of GO enrichment analysis showing significantly enriched terms (adjusted $P < 0.05$) in biological process, molecular function, and cellular component categories for the three sub-clusters. Bubble size indicates the number of enriched genes; color intensity reflects enrichment significance. (B, D, F) Bar plots of Reactome pathway enrichment displaying the top 20 significantly enriched pathways (adjusted $P < 0.05$) for each sub-cluster. Bar length indicates the number of enriched genes.

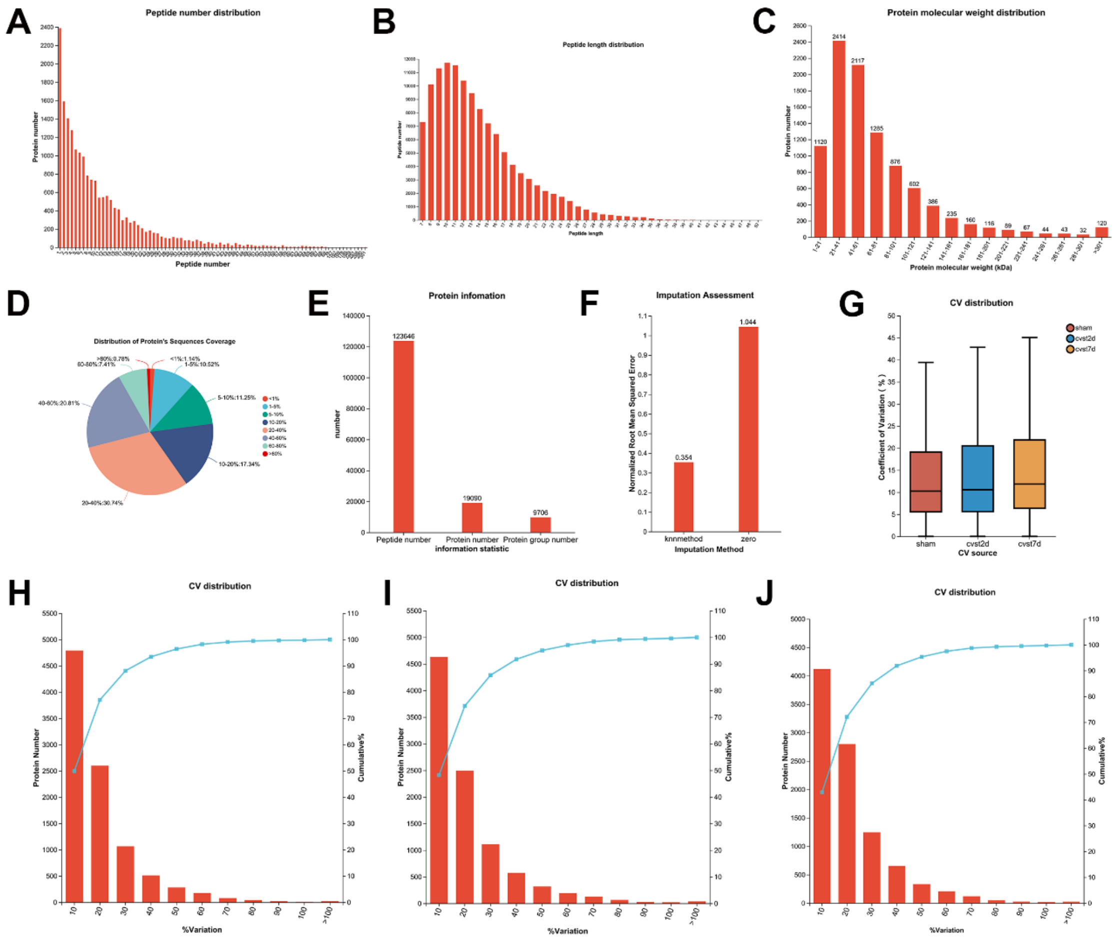

Supplementary Figure S5. Quality control of proteomic data. (A) Peptide-coverage distribution: number of proteins identified by a given number of peptides. (B) Peptide-length distribution: number of identified peptides across different amino-acid lengths. (C) Protein molecular-weight distribution: number of identified proteins within indicated molecular-weight ranges. (D) Pie chart showing the distribution of protein sequence coverage. (E) Summary statistics of identified peptides, proteins, and protein groups. (F) Comparison of relative standard deviations after k-NN imputation versus zero-value imputation for missing data. (G) Box-plot showing inter-group CV distribution of protein quantification values. (H–J) Intra-group CV distribution (histogram) and cumulative percentage curve (line) for Sham (H), CVST 2d (I), and CVST 7d (J) groups.

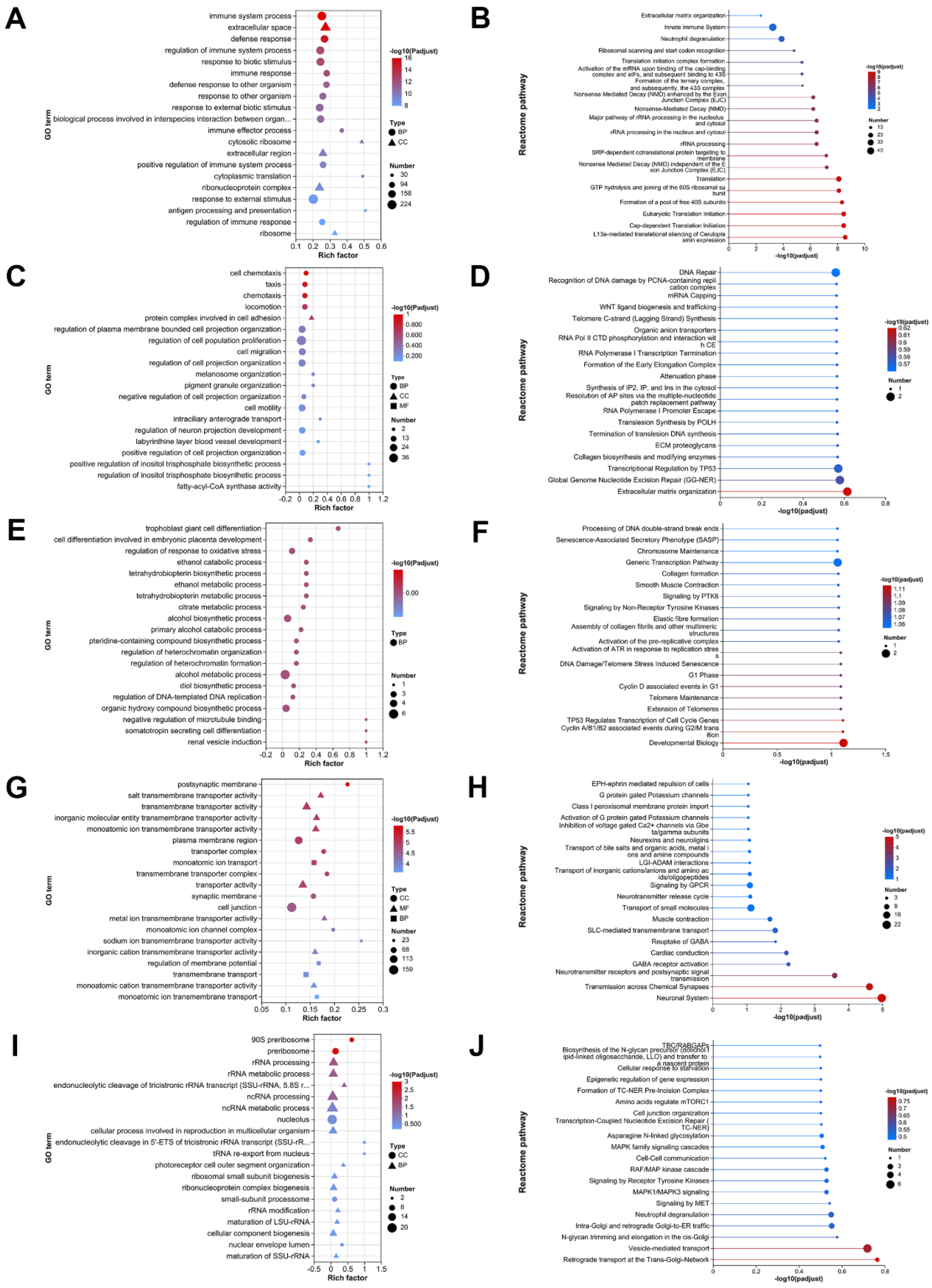

Supplementary Figure S6. Functional enrichment analysis of five protein sub-classes. (A, C, E, G, I) Bubble plots of GO enrichment; (B, D, F, H, J) bar plots of Reactome pathway enrichment.

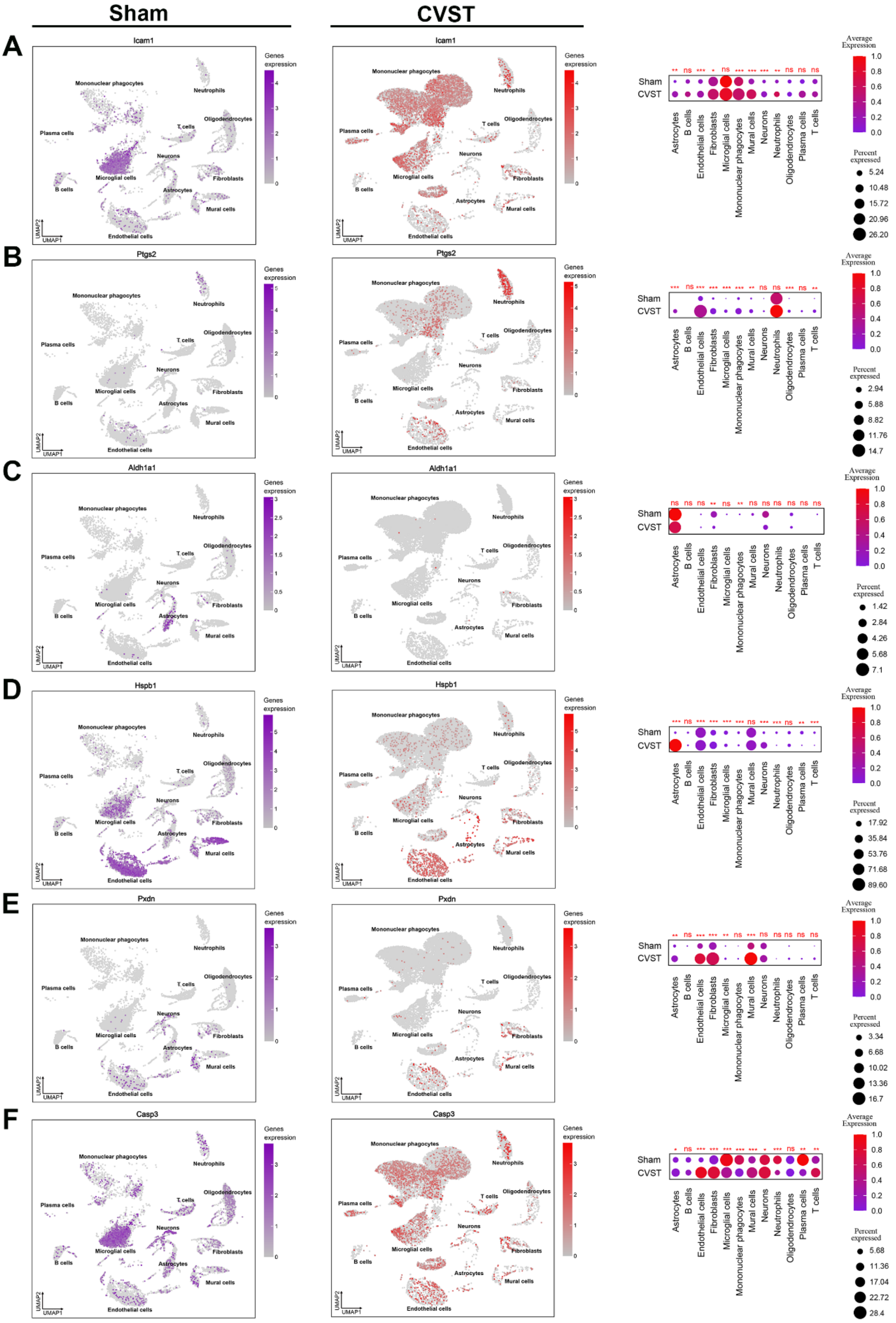

Supplementary Figure S7. Cell-type-specific expression of six core genes. (A–F) show the expression levels of Icam1 (A), Ptgs2 (B), Aldh1a1 (C), Hspb1 (D), Pxdn (E), and Casp3 (F) across 12 cell types in Sham and CVST groups. Significance: ***P < 0.001, **P < 0.01, *P < 0.05; ns, not significant.

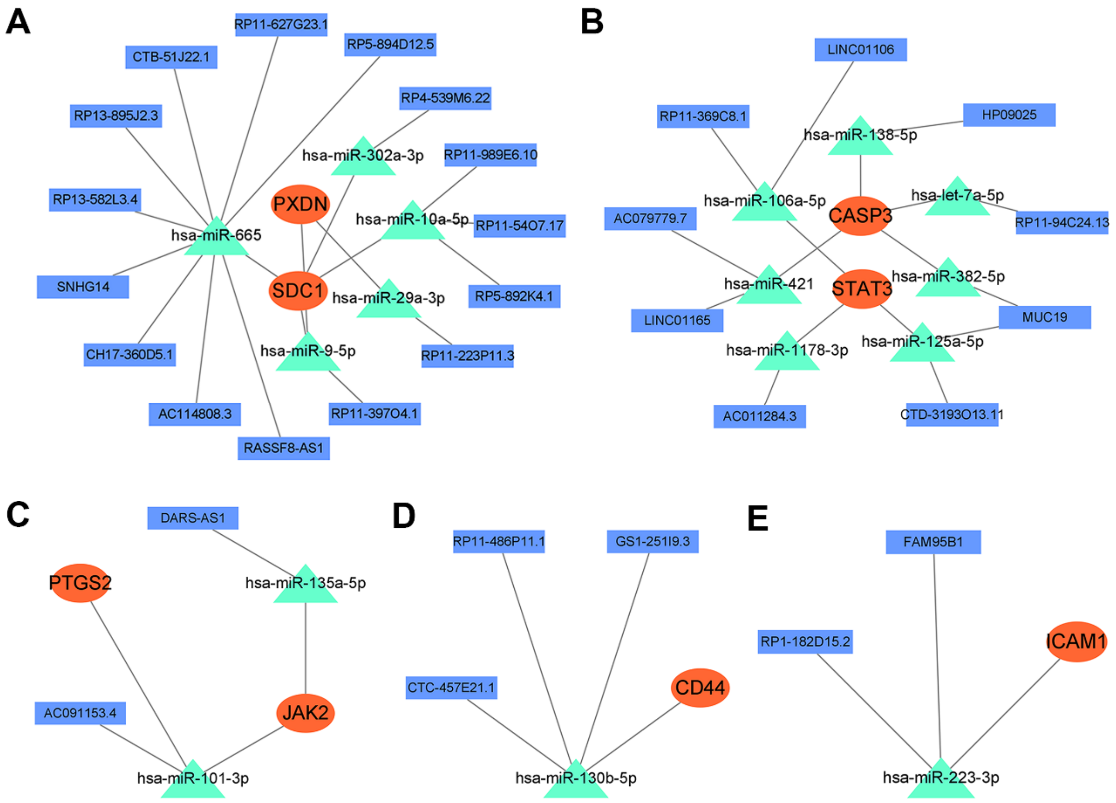

Supplementary Figure S8. ceRNA regulatory network of core genes. Orange nodes: core genes; cyan nodes: miRNAs; blue nodes: lncRNAs.
