# Supplementary material for: Integrated Multi-Omics Identifies Core Molecular Targets in Cerebral Venous Sinus Thrombosis-Induced Brain Injury
Source: Biomedicines. 2026 Jul 16;14(7):1594. doi: 10.3390/biomedicines14071594 (PMC13406610; doi:10.3390/biomedicines14071594)
Supplement: Supplementary file 1 [file biomedicines-14-01594-s001.zip › Supplementary Materials and Methods.pdf]

Supplementary Materials and Methods

1 Supplementary Materials and Methods 1 (Transcriptomics)

1. 1 RNA extraction

Total RNA was extracted from the tissue using MJZol total RNA extraction kit Reagent according the manufacturer's instructions. Then RNA quality was determined by 5300 Bioanalyser (Agilent) and quantified using the ND-2000 (NanoDrop Technologies). Only high-quality RNA sample (OD260/280 = 1.8~2.2, OD260/230 ≥ 2.0, RQN ≥ 6.5, 28S:18S ≥ 1.0, >1μg) was used to construct sequencing library.

1.2 Library preparation and Sequencing

RNA purification, reverse transcription, library construction and sequencing were performed at Shanghai Majorbio Bio-pharm Biotechnology Co., Ltd. (Shanghai, China) according to the manufacturer's instructions . The RNA-seq transcriptome librariy was prepared following Illumina® Stranded mRNA Prep, Ligation (San Diego, CA) using 1μg of total RNA. Shortly, messenger RNA was isolated according to polyA selection method by oligo(dT) beads and then fragmented by fragmentation buffer firstly. Secondly double-stranded cDNA was synthesized using a SuperScript double-stranded cDNA synthesis kit (Invitrogen, CA) with random hexamer primers. Then the synthesized cDNA was subjected to end-repair, phosphorylation and adapter addition according to library construction protocol. Libraries were size selected for cDNA target fragments of 300 bp on 2% Low Range Ultra Agarose followed by PCR amplified using Phusion DNA polymerase (NEB) for 15 PCR cycles. After quantified by Qubit 4.0, the sequencing library was performed on NovaSeq X Plus platform(PE150) using NovaSeq Reagent Kit. (NovaSeq) OR the sequencing library was performed on DNBSEQ-T7 platform(PE150) using DNBSEQ-T7RS Reagent Kit (FCL PE150) version 3.0.

2 Supplementary Materials and Methods 2 (Proteomics)

2.1 Total protein extraction

The samples were taken out in the frozen state and put on ice. The samples were suspended in protein lysis buffer (8M urea,1% SDS) which included appropriate

protease inhibitor to inhibit protease activity and the mixture were treated by high-flux tissue grinding machine for 3 times, 40 s each. Then the mixture was incubated on ice for 30 min, during which was vortex mixed for 5-10 s every 5 min. After centrifugation at 16000g at 4°C for 30min, the concentration of protein from the supernatant collected was determined by Bicinchoninic acid (BCA) method by BCA Protein Assay Kit (Thermo Scientific). Protein quantification was performed according to the kit protocol.

2.2 Protein digestion

100 μg protein re-suspended with Triethylammonium bicarbonate buffer (TEAB) which with the final concentration of 100mM. The mixture was reduced with Tris(2-carboxyethyl)phosphine (TCEP) which with the final concentration of 10mM at 37 °C for 60min and alkylated with iodoacetamide (IAM) which with the final concentration of 40mM at room temperature for 40min in darkness. After centrifugation at 10000g at 4°C for 20min, the pellet was collected, which re-suspended with 100μl Triethylammonium bicarbonate buffer(TEAB) which with the final concentration of 100mM. Trypsin was added at 1:50 trypsin- to-protein mass ratio and incubated at 37 °C overnight.

2.3 Peptide desalting and quantification

After trypsin digestion, the peptides were drained by vacuum pump. Then, the enzymatically drained peptides were re-solubilized with 0.1% trifluoroacetic acid (TFA), and the peptides were desalted with HLB and drained by vacuum concentrator. Finally, the peptides were quantified using the Thermo Fisher Scientific Peptide Quantification Kit (item #23275).

2.4 DIA mass detection

The equivalent peptides were redissolved in spectrometry loading buffer (2% ACN with 0.1% formic acid) which included appropriate iRT peptide which was used to calibrate retention time and analyzed by an EASY nLC-1200 system (Thermo, USA) coupled with a Q Exactive HF-X quadrupole orbitrap mass spectrometer (Thermo, USA) at Majorbio Bio-Pharm Technology Co. Ltd. (Shanghai, China). Briefly, the C18-reversed phase column (75 μm x 25 cm , Thermo ,USA) as

equilibrated with solvent A (2% ACN with 0.1% formic acid) and solvent B (80% ACN with 0.1% formic acid). The flow rate of 300 nL/min. The peptides were eluted using the following gradient: 0-70 min, 5%-23% B; 70-90 min, 23%−29% B; 90-100 min, 29%-38% B; 100-102 min, 38%-48% B; 102-103 min, 48%-100% B; 103-120 min, maintain 100% B.

The Q Exactive HF-X instrument was operated in the data-independent acquisition mode (DIA)(1) to automatically switch between full scan MS and MS/MS acquisition. The survey of full scan MS spectra (m/z 300-1500) was acquired, then the all-precursor ions were selected into collision cell for fragmentation by HCD. DIA was performed with a variable isolation window, each window overlapped by 1 m/z, there were 40 windows in total.

2.5 DIA data analysis

DIA raw data was imported into Spectronaut™ software (Version 14) for library search analysis. The database used in this study is UniProt. Retention times were corrected by iRT and 6 peptides per protein and 3 daughter ions per peptide were selected for quantitative analysis. The parameters are as follows up : Protein FDR ≤ 0.01, Peptide FDR ≤ 0.01, Peptide Confidence ≥ 99%, XIC width ≤ 75ppm(2, 3). The shared peptides and modified peptides were excluded, and the peak areas were calculated and summed to give the quantitative results.

3 Supplementary Materials and Methods 3 (Single-cell Transcriptome Sequencing Analysis)

3.1 Tissue dissociation and preparation

The fresh tissues were stored in the sCelLive™ Tissue Preservation Solution (Singleron) on ice after the surgery within 30 mins. The specimens were washed with Hanks Balanced Salt Solution (HBSS) for three times, minced into small pieces, and then digested with 3 mL sCelLive™ Tissue Dissociation Solution (Singleron) by Singleron PythoN™ Tissue Dissociation System at 37 °C for 15 min. The cell suspension was collected and filtered through a 40-micron sterile strainer. Afterwards, the GEXSCOPE® red blood cell lysis buffer (RCLB, Singleron) was added, and the mixture[Cell: RCLB=1:2 (volume ratio)] was incubated at room temperature for 5-8

min to remove red blood cells. The mixture was then centrifuged at 300 × g 4 °C for 5 mins to remove supernatant and suspended softly with PBS. Finally, the samples were stained with Trypan Blue and the cell viability was evaluated microscopically.

3.2 RT & Amplification & Library Construction

Single-cell suspensions (2×10^5 cells/mL) with PBS (HyClone) were loaded onto microwell chip using the Singleron Matrix® Single Cell Processing System. Barcoding Beads are subsequently collected from the microwell chip, followed by reverse transcription of the mRNA captured by the Barcoding Beads and to obtain cDNA, and PCR amplification. The amplified cDNA is then fragmented and ligated with sequencing adapters. The scRNA-seq libraries were constructed according to the protocol of the GEXSCOPE® Single Cell RNA Library Kits (Singleron)(4). Individual libraries were diluted to 4 nM, pooled, and sequenced on Illumina novaseq 6000 with 150 bp paired end reads.

Reference

1. He Y, Yu S, Liu S, Tian H, Yu C, Tan W, et al. Data-Independent Acquisition Proteomics Unravels the Effects of Iron Ions on Coronatine Synthesis in Pseudomonas syringae pv. tomato DC3000. Front Microbiol. 2020;11:1362.doi:10.3389/fmicb.2020.01362.
2. Zhu T, Zhu Y, Xuan Y, Gao H, Cai X, Piersma SR, et al. DPHL: A DIA Pan-human Protein Mass Spectrometry Library for Robust Biomarker Discovery. Genomics, Proteomics & Bioinformatics. 2019;18(2).doi:10.1016/j.gpb.2019.11.008.
3. Xu M, Deng J, Xu K, Zhu T, Han L, Yan Y, et al. In-depth serum proteomics reveals biomarkers of psoriasis severity and response to traditional Chinese medicine. Theranostics. 2019;9(9):2475-88.doi:10.7150/thno.31144.
4. Burak D, Jin-Young C, Kerou Z, William D, Durga T, Marcus B, et al. scFTD-seq: freeze-thaw lysis based, portable approach toward highly distributed single-cell 3' mRNA profiling. Nucleic acids research. 2019;47(3):e16.doi:10.1093/nar/gky1173.
